# Improved chloroplast energy balance during water deficit enhances plant growth: more crop per drop

**DOI:** 10.1093/jxb/erx474

**Published:** 2017-12-21

**Authors:** Keshav Dahal, Greg C Vanlerberghe

**Affiliations:** Department of Biological Sciences and Department of Cell and Systems Biology, University of Toronto Scarborough, Toronto, Ontario, Canada

**Keywords:** CO_2_ assimilation, energy balance, growth, mitochondrial alternative oxidase, photosynthesis, respiration, thylakoid membrane proton circuit, water deficit

## Abstract

The non-energy-conserving alternative oxidase (AOX) respiration of plant mitochondria is known to interact with chloroplast photosynthesis. This may have consequences for growth, particularly under sub-optimal conditions when energy imbalances can impede photosynthesis. This hypothesis was tested by comparing the metabolism and growth of wild-type *Nicotiana tabacum* with that of AOX knockdown and overexpression lines during a prolonged steady-state mild to moderate water deficit. Under moderate water deficit, the AOX amount was an important determinant of the rate of both mitochondrial respiration in the light and net photosynthetic CO_2_ assimilation (*A*) at the growth irradiance. In particular, AOX respiration was necessary to maintain optimal proton and electron fluxes at the chloroplast thylakoid membrane, which in turn prevented a water-deficit-induced biochemical limitation of photosynthesis. As a result of differences in *A*, AOX overexpressors gained more biomass and knockdowns gained less biomass than wild-type during moderate water deficit. Biomass partitioning also differed, with the overexpressors having a higher percentage, and the knockdowns having a lower percentage, of total above-ground biomass in reproductive tissue than wild-type. The results establish that improving chloroplast energy balance by using a non-energy-conserving respiratory electron sink can increase photosynthesis and growth during prolonged water deficit.

## Teaser

Mitochondrial alternative oxidase respiration aids in balancing the thylakoid membrane proton circuit, thus improving both photosynthesis and growth during a prolonged water deficit.

## Introduction

Photosynthesis and respiration represent the core of carbon and energy metabolism, and hence are key determinants of growth (Stitt *et al.*, 2010; Millar *et al.*, 2011). However, these metabolic systems may experience energy imbalances, such as a mismatch between supply and demand for ATP and/or NAD(P)H. Such imbalances may have consequences for overall plant performance (Hüner *et al.*, 1998; Wilson *et al.*, 2006; De Block and Van Lijsebettens, 2011). In photosynthesis, the absorption of light energy by chloroplast thylakoid pigments drives the generation of ATP and NADPH, which are used by the Calvin cycle to produce triose phosphates through the assimilation of CO_2_. However, owing to factors such as a highly variable light environment and availability of CO_2_, photosynthesis is prone to energy imbalances (Kramer and Evans, 2011). For example, reduced availability of CO_2_ during water deficit, due to stomatal closure, reduces the consumption of ATP and NADPH being generated by the thylakoid reactions (Lawlor, 2002; Flexas *et al.*, 2004).

In plant mitochondria, the respiratory electron transport chain is branched, such that electrons from ubiquinol can flow to cytochrome (cyt) oxidase (via Complex III and cyt *c*) or the alternative oxidase (AOX) (Finnegan *et al.*, 2004; Plaxton and Podestá, 2006; Noctor *et al.*, 2007; Millar *et al.*, 2011). Electron flow to AOX bypasses two of three sites of proton translocation across the inner mitochondrial membrane. While this reduces the ATP yield of the respiratory chain, it also allows NAD(P)H turnover to continue, even when energy charge (ATP/ADP) is high (Vanlerberghe *et al.*, 1995). Such flexibility may prevent an over-reduction of respiratory chain components that can otherwise result in the generation of reactive oxygen and nitrogen species (Maxwell *et al.*, 1999; Cvetkovska and Vanlerberghe, 2012; Alber *et al.*, 2017).

The impact of AOX respiration on overall growth remains poorly understood (Vanlerberghe, 2013). In theory, AOX could negatively impact growth since growth requires ATP and AOX reduces the ATP yield of respiration. On the other hand, this energy cost might be outweighed by beneficial impacts of AOX, perhaps related to maintaining energy balance. Several studies, including those using mutant or transgenic lines, have investigated the link between AOX respiration and growth. These studies have yielded conflicting results, with both positive and negative impacts of AOX on growth being reported (Millar *et al.*, 1998; Millenaar *et al.*, 2001; Fiorani *et al.*, 2005; Sieger *et al.*, 2005; Florez-Sarasa *et al.*, 2007; Mathy *et al.*, 2010; Skirycz *et al.*, 2010; Chai *et al.*, 2012; Zidenga *et al.*, 2012). Hence, AOX remains a component of primary metabolism whose impact on growth remains elusive (Gifford, 2003; Noctor *et al.*, 2007; Lawlor and Tezara, 2009; Amthor, 2010; Nunes-Nesi *et al.*, 2011). Since AOX gene expression and protein amount often increase in response to abiotic and biotic stresses, these might be conditions where the pathway is particularly beneficial (Clifton *et al.*, 2006). Computational models predict increased AOX respiration with increases in irradiance, a prediction based on the ability of AOX to aid in the maintenance of energy balance during photosynthesis (Buckley and Adams, 2011; Cheung *et al.*, 2015; Nikoloski *et al.*, 2015).

The chloroplast itself is well-equipped with mechanisms to balance ATP and NAD(P)H supply by the thylakoid reactions with ATP and NADPH use by the Calvin cycle and other chloroplast metabolism (Oelze *et al.*, 2008; Kramer and Evans, 2011; Foyer *et al.*, 2012; Schӧttler *et al.*, 2015; Wobbe *et al.*, 2016; Foyer *et al.*, 2017). In thylakoid membranes, transfer of light energy to photosystem II (PSII) and photosystem I (PSI) drives electron transport to generate NADPH. Electron transport is coupled to proton translocation from stroma to lumen, generating a proton motive force (pmf) across the thylakoid membrane with both chemical potential (∆pH) and electrical potential (∆ψ) components. This pmf is used by the thylakoid membrane-localized ATP synthase to drive the synthesis of ATP. The total pmf can be composed of variable fractions of ∆pH and ∆ψ, both of which are equally effective at driving ATP synthesis. However, ∆pH also has important roles in the regulation of photosynthesis and maintenance of energy balance. A lower lumen pH supports the generation of non-photochemical energy quenching (NPQ) and slows electron flux through the cyt *b*_6_*f* complex (Cruz *et al.*, 2005; Foyer *et al.*, 2012; Tikhonov, 2014). Given this dual role of lumen protons to both support ATP synthesis and regulate photosynthesis, the proton circuit between stroma and lumen must be tightly and flexibly regulated, in order to optimize photosynthesis in response to changing metabolic and environmental conditions. It is thus a central regulatory feature of chloroplast metabolism (Kramer *et al.*, 2003; Cruz *et al.*, 2005; Kohzuma *et al.*, 2009; Tikhonov, 2013; Armbruster *et al.*, 2017; Shikanai and Yamamoto, 2017). Nonetheless, it is recognized that extra-chloroplast metabolism, including mitochondrial metabolism, is also required to optimize photosynthesis (Krӧmer, 1995; Hoefnagel *et al.*, 1998; Raghavendra and Padmasree, 2003; Noguchi and Yoshida, 2008; Taniguchi and Miyake, 2012; Tcherkez *et al.*, 2012; Gardestrӧm and Igamberdiev, 2016). The details of these beneficial chloroplast–mitochondrial interactions are less well understood.

We previously showed that, in response to a rapid onset of water deficit stress, the severity of which increased continually over time, the AOX amount in *Nicotiana tabacum* leaf was a strong determinant of the rate of respiration in the light (*R*_L_) (Dahal *et al.*, 2014; Dahal and Vanlerberghe, 2017). In turn, *R*_L_ strongly influenced short-term photosynthetic performance. Here, we show that increased AOX respiration is also an important acclimation to a prolonged steady-state moderate water deficit. We show that AOX respiration improves net CO_2_ assimilation rate (*A*) by promoting energy balance in the chloroplast, in particular by staving off changes in the thylakoid proton circuit that otherwise culminate in a biochemical limitation of photosynthesis. Finally, we show that the long-term increase in *A* promoted by AOX respiration improves growth under water deficit.

## Materials and methods

### Plant material, growth conditions, and experimental design

Wild-type (WT) and transgenic *Nicotiana tabacum* cv. Petit Havana were used. Plant lines B7 and B8 have elevated amounts of AOX protein due to the presence of an *AOX1a* transgene driven by a constitutive promoter, while plant lines RI9 and RI29 have suppressed amounts of AOX protein due to the presence of an *AOX1a* RNA interference construct. All are independent transgenic lines homozygous for their transgene (Wang *et al.*, 2011; Wang and Vanlerberghe, 2013; Cvetkovska *et al.*, 2014; Dahal and Vanlerberghe, 2017). A key objective was to subject plants to a reasonably steady-state and moderate degree of water deficit throughout their growth period. This would allow a comparison of the plant lines under conditions where they had become well acclimated to a long-term moderate water deficit (Harb *et al.*, 2010).

Seeds were germinated in vermiculite at room temperature and low light. For each independent experiment, tiny seedlings (16 d after sowing) were transplanted into four large rectangular pots (74 cm long, 23 cm wide, 23 cm deep). Each pot contained five seedlings (one each of WT, B8, B7, RI9, and RI29) equally spaced along the length of the pot. The order of plant lines along the length of the pot was randomized across the four pots. Each pot contained an equivalent weight of dry growing medium (8.8 kg) that was saturated to field capacity with water (12.1 litres) prior to transplanting. The growing medium had four parts soil (Pro-mix BX, Premier Horticulture, Rivière-du-Loup, Canada) and one part vermiculite. The pots were placed in growth chambers (model PGC-20, Conviron, Winnipeg, Canada) with 16 h photoperiod, temperature of 28 °C/22 °C (light/dark), relative humidity of 60% and photosynthetic photon flux density (PPFD) of 700 µmol m^−2^ s^−1^ (700 PPFD).

Preliminary experiments with WT plants established a watering schedule (see Supplementary Fig. S1 at *JXB* online) that allowed the seedlings to become established, while also consistently generating plants in which the relative water content (RWC) of leaf 5 was approximately 82% by day 23 in the growth chamber (Supplementary Fig. S2A). Since well-watered tobacco normally maintain a RWC closer to 90%, the day 23 plants were experiencing what we have previously characterized as mild water deficit (Dahal and Vanlerberghe, 2017). Following the established watering schedule then resulted in a gradual decline in RWC to approximately 71% by day 33, characterized as moderate water deficit (Dahal and Vanlerberghe, 2017). Following this, the watering schedule generated plants where RWC remained relatively stable (fluctuating between 66 and 72%) through day 50 (Supplementary Fig. S2A). Throughout this time course, leaf RWC correlated closely with soil water content (Supplementary Fig. S2B). In experiments comparing the different plant lines, an identical watering schedule was used, and most of the physiological and biochemical analyses (see below) were performed at days 23, 33, 39, and 45 after transplanting. Since some of these analyses required destructive sampling, one of the four pots was used at each time point, within each independent experiment. In these experiments, soil water content averaged 86% on day 23, and then ranged from 57 to 63% on the other days (Supplementary Fig. S2D).

### Gas exchange and Chl *a* fluorescence

Leaf CO_2_ exchange and Chl *a* fluorescence from PSII were measured in the growth chamber using a portable system (GFS-3000; Heinz Walz, Effeltrich, Germany). Gas exchange data were used to calculate *A*, transpiration rate (*T*) and stomatal conductance (*g*_s_) (von Caemmerer and Farquhar, 1981; Farquhar and Sharkey, 1982). Instantaneous leaf water use efficiency (iWUE) was calculated as *A*/*T* (Condon *et al.*, 2004; Bernacchi and VanLoocke, 2015). Gas exchange was also used to evaluate respiration. Respiration rate in the dark (*R*_D_) was measured following a 30 min dark pre-incubation. *R*_L_ was estimated by the Kok method as previously described (Dahal *et al.*, 2014).

Simultaneous gas exchange and Chl *a* fluorescence analyses followed a dark pre-incubation of 30 min. Minimum fluorescence (*F*_o_), maximal fluorescence in the dark-adapted leaf (*F*_m_) or light-adapted leaf (*F*_m_′), steady state fluorescence in the light-adapted leaf (*F*_s_), and minimal fluorescence in the light-adapted leaf (*F*_o_′) were used to calculate photosynthetic parameters (Maxwell and Johnson, 2000). The maximal quantum yield of PSII (*F*_v_/*F*_m_) was calculated as (*F*_m−_*F*_o_)/*F*_m_ and the effective quantum yield or operating efficiency of PSII (Φ_PSII_) was calculated as (*F*_m_′−*F*_s_)/*F*_m_′ (Genty *et al.*, 1989). The rate of linear electron transport (LET) through PSII (ETR) was calculated as ETR=(Φ_PSII_×PPFD×0.5 × 0.84) where 0.84 and 0.5 represent estimates that leaves absorb 84% of incident photons and that 50% of these are absorbed by PSII (Yamori *et al.*, 2011*a*). Photochemical energy quenching (qP) was calculated using the puddle model where qP=(*F*_m_′−*F*_s_)/(*F*_m_′−*F*_o_′) (Kramer *et al.*, 2004). The fraction of closed (reduced) PSII reaction centers, known as excitation pressure, was calculated as 1−qP. NPQ, a measure of heat dissipation of absorbed light energy, was calculated as (*F*_m−_*F*_m_′)/*F*_m_′ (Maxwell and Johnson, 2000). Light response curves were measured at intervals over the range of 0–2000 PPFD (from low to high PPFD, 6 min at each irradiance) and CO_2_ concentration of 400 μmol mol^−1^.

### Leaf absorption spectroscopy

A DUAL-PAM-100 measuring system (Heinz Walz) equipped with DUAL-E and DUAL-DB modules were used to simultaneously measure PSI absorbance and PSII fluorescence. Absorbance changes were used to estimate the rate of electron transport through PSI (Klughammer and Schreiber, 2008), while fluorescence changes were used to estimate ETR through PSII, as described above. Rates of cyclic electron transport (CET) around PSI were then estimated by subtracting the measured ETR from the measured rate of electron transport through PSI (Johnson, 2011). CET was measured at intervals over the range of 0–2000 PPFD (from low to high PPFD, 6 min at each irradiance) and CO_2_ concentration of 400 μmol mol^−1^.

A Dual-PAM-100 measuring system equipped with the emitter module DUAL-EP515 and the detector module DUAL-DP515 was used to measure the 550–515 nm absorbance difference signal (Schreiber and Klughammer, 2008). This signal provides a linear measure of thylakoid membrane ∆ψ due to a ∆ψ-induced shift in the absorption spectrum of thylakoid pigments, known as the electrochromic shift (ECS). A dark-interval relaxation kinetics (DIRK) analysis of the ECS signal during a light-to-dark transition was used to estimate several parameters related to thylakoid proton flux (Cruz *et al.*, 2005; Baker *et al.*, 2007). These measurements rely upon the principal that, under steady-state conditions, proton influx to the lumen due to electron transport is balanced by proton efflux from the lumen through ATP synthase. As previously described, the total rapid decay in the ECS signal over the initial approximately 300 ms of dark (ECS_t_) is an estimate of the total light-induced pmf (Cruz *et al.*, 2005; Baker *et al.*, 2007). A firstorder fit of this rapid exponential decay provides a time constant (τ_ECS_) inversely proportional to the proton conductivity of ATP synthase (*g*_H+_) calculated as *g*_H+_=1/τ_ECS_. The relative rate of proton flux across the thylakoid membrane (*v*_H+_) is then estimated as ECS_t_/τ_ECS_ (Cruz *et al.*, 2005; Baker *et al.*, 2007). Beyond 300 ms and up to approximately 120 s after the light–dark transition, an inverse ECS signal develops due to continued proton efflux. This signal is exploited to partition ECS_t_ into two components, ECS_inv_ and ECS_ss_, corresponding respectively to the ∆pH and ∆ψ components of the total light-induced pmf, as outlined previously (Cruz *et al.*, 2005; Baker *et al.*, 2007). For the DIRK analysis, plants were dark-adapted for 1 h, followed by illumination (700 or 1600 PPFD) for 10 min prior to the light–dark transition. It should be noted that the ECS signal and its meaning remain controversial due to the possibility of confounding overlapping absorbance signals (Johnson and Ruban, 2014).

### Biochemical, growth, and other analyses

Leaf protein amounts were estimated by immunoblot analyses. Protein extraction, protein separation by SDS-PAGE, transfer of proteins to nitrocellulose, and incubation with antibodies were performed as before (Dahal *et al.*, 2014). Primary antibodies (Agrisera, Vännäs, Sweden) were diluted 1000–5000-fold, and raised against the following proteins: PsbA (D1 reaction center protein of PSII), PsbS (a PSII-associated sensor of lumen pH necessary for NPQ induction), PsaA (a reaction center protein of PSI), Cyt_*f*_ (the *c*-type cyt subunit of the Cyt *b*_6_*f* complex), AtpΒ (the Β-subunit of the F_1_ catalytic subcomplex of ATP synthase), RbcS (small subunit of Rubisco) and AOX. Secondary antibody was detected by chemiluminescence (Clarity Western ECL Substrate, Bio-Rad Laboratories, Mississauga, Canada) and X-ray film. The signals were quantified using an image analysis system (Chemidoc XRS+ with Image Lab Software v.3.0, Bio-Rad).

To evaluate growth, the dry weight (DW) of different plant parts was determined following oven drying (48 h, 80 °C). Leaf area was measured with an area meter (Model LI-3000C, LiCor Biosciences, Lincoln, NE, USA). Plant height was measured from stem base to shoot apex. In most cases, growth was analysed at days 23, 33, 39, and 45 of the experiments described above. However, in some cases, the moderate water deficit was extended until day 92, by which time the plants had fully senesced and all seed pots had fully dried. These plants were used to determine seed pod number and weight, and the seed was tested using a germination assay. Germination was tested on agar plates (containing Murashige and Skoog salts, vitamins and 1% sucrose) incubated under constant conditions (75 PPFD, 25 °C) for 7 d.

Total leaf Chl and protein amounts were determined as previously described (Dahal *et al.*, 2014). Leaf RWC was determined as before (Wang and Vanlerberghe, 2013) and also used to determine specific leaf weight. Soil water content (% saturation) at different times during an experiment was determined by measuring the pot weight and comparing it to the pot weight at the beginning of the experiment, when the soil was at field capacity and defined as 100% saturated. Statistical analyses (two-way or one-way ANOVA, followed by a Bonferroni post-test to compare all plant lines within a treatment) were performed using Prism 5.0 (GraphPad Software).

## Results

### AOX is an important determinant of *R*_L_ during a prolonged water deficit

On all days, there were no differences in leaf 5 RWC across plant lines, indicating that the differences in the AOX amount across lines had no impact on leaf water status (see Supplementary Fig. S2C). There was a moderate decline in leaf RWC in all lines over the course of the experiment. For example, RWC in the WT was 82% on day 23, 71% on day 33, 67% of day 39 and 70% on day 45. This allowed us to compare the plants under both mild (day 23) and moderate (days 33–45) water deficit conditions, while also maintaining a relatively constant moderate water deficit over an extended period. This allowed us to reveal differences in long-term processes such as growth across the plant lines (see below).

There were gradual changes in several other leaf 5 parameters over the course of the experiment. In WT plants, the mean value of total Chl and total protein declined 24% and 26%, respectively, while the Chl *a*/*b* ratio and specific leaf weight increased 16% and 37%, respectively, between days 23 and 45 (see Supplementary Fig. S3A, C, D). On day 23, these parameters were similar among WT, AOX knockdown, and AOX overexpression plants. However, by day 45, a pattern emerged whereby total Chl and total protein were modestly higher (by 12% and 11%, respectively) in the overexpressors (average of two lines) and modestly lower (by 16% and 15%, respectively) in the knockdowns, compared with WT. By day 45, the mean Chl *a*/*b* ratio was 9% lower in the overexpressors and 18% higher in the knockdowns, compared with WT (Supplementary Fig. S3B). However, there was no difference in leaf 5 specific weight (Supplementary Fig. S3D) or size (Supplementary Fig. S4) across plant lines over the course of the experiment.

**Fig. 1.**
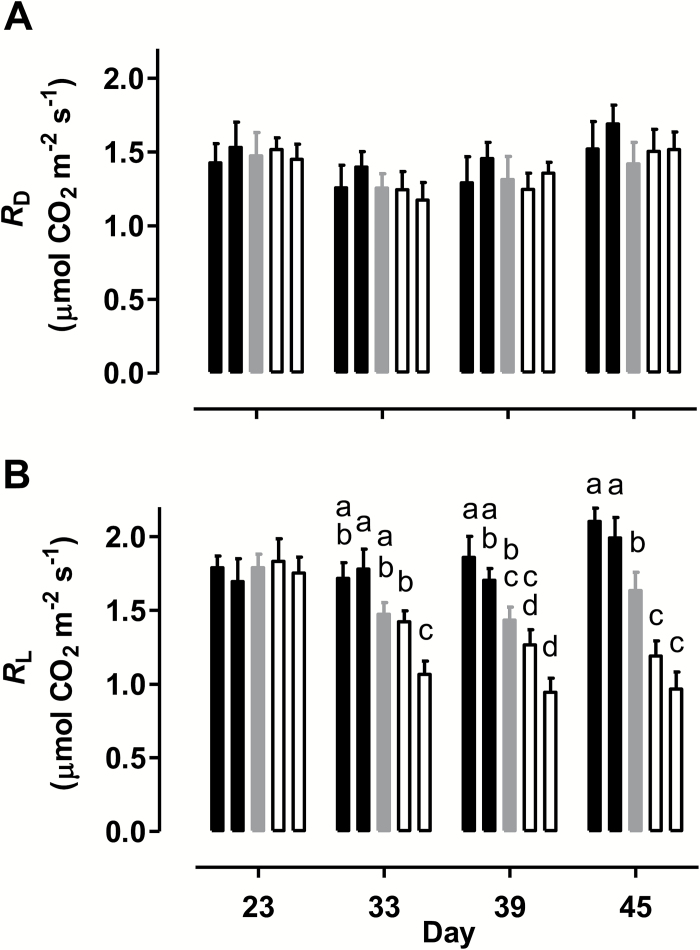
Respiration rate of tobacco leaf at different times during a prolonged water deficit. (A) *R*_D_. (B) *R*_L_. Data are shown for WT (gray bar), AOX overexpressors (B8, left closed bar; B7, right closed bar), and AOX knockdowns (RI9, left open bar; RI29, right open bar). Data are the mean±SE of three independent experiments (*n*=3). Within each data set, plant lines not sharing a common letter are significantly different from one another (*P*<0.05). In data sets without letters, there are no significant differences across plant lines.

There were no significant differences in leaf 5 *R*_D_ across plant lines on any of the days examined (Fig. 1A). In all plant lines, there was perhaps a small decline in *R*_D_ between day 23 and 33, followed by a recovery of the rate through days 39 and 45. There were also no differences in *R*_L_ across plant lines on day 23 (Fig. 1B). However, by day 33, a pattern emerged where *R*_L_ was highest in the AOX overexpressors and lowest in the AOX knockdowns, with WT showing an intermediate rate. These differences in *R*_L_ were exaggerated further by day 39 and again by day 45. On day 45, the overexpression lines averaged 25% higher *R*_L_ and the knockdowns averaged 34% lower *R*_L_ than in WT (Fig. 1B).

### AOX respiration improves *A* during a prolonged water deficit

**Fig. 2.**
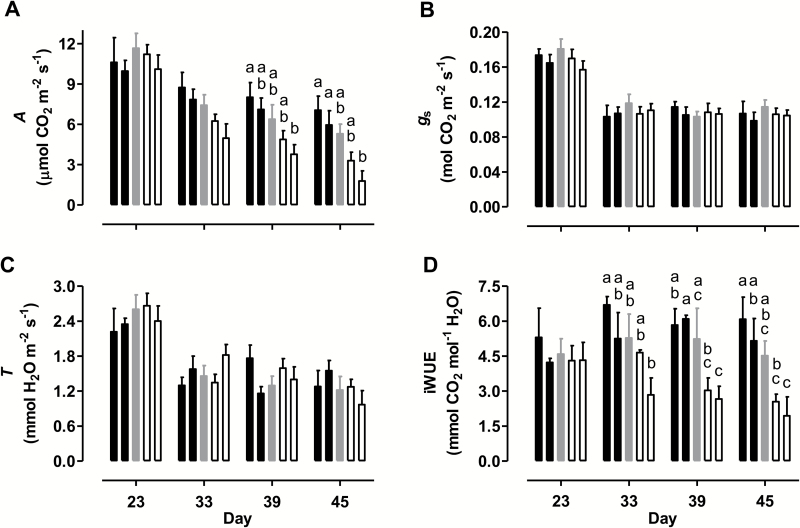
Photosynthesis-related parameters of tobacco leaf at different times during a prolonged water deficit and measured at the growth irradiance. (A) *A*. (B) *g*_s_. (C) *T*. (D) iWUE. Data are shown for WT (gray bars), AOX overexpressors (B8, left closed bar; B7, right closed bar), and AOX knockdowns (RI9, left open bar; RI29, right open bar). Data are the mean±SE of three independent experiments (*n*=3). Within each data set, plant lines not sharing a common letter are significantly different from one another (*P*<0.05). In data sets without letters, there are no significant differences across plant lines.

In WT plants, the mean *A* of leaf 5, measured at growth irradiance, declined from 11.7 to 7.4 μmol CO_2_ m^−2^ s^−1^ between days 23 and 33 (Fig. 2A). A further small decline occurred by day 39 (to 6.4 μmol m^−2^ s^−1^) and then again by day 45 (to 5.3 μmol m^−2^ s^−1^). Under mild water deficit (day 23), the five plant lines maintained similar mean *A*. However, by day 33 a pattern emerged where *A* was slightly higher in the overexpressors and slightly lower in the knockdowns, compared with WT. This pattern was exaggerated further by day 39 and again by day 45 (Fig. 2A). On day 45, the overexpressors averaged 23% higher *A*, and the knockdowns averaged 52% lower *A* than WT, when measured at the growth irradiance. Light response curves showed similar dramatic differences in *A* across the plant lines over time (see Supplementary Fig. S5).

Leaf 5 *g*_s_ and *T* measured at growth irradiance did not differ across plant lines on any of the days examined (Fig. 2B, C). In all lines, these two parameters declined noticeably between days 23 and 33, and then remained relatively constant for the remainder of the experiment. In WT plants, the mean iWUE of leaf 5 measured at growth irradiance was relatively stable (ranging from 4.5 to 5.3 mmol CO_2_ mol^−1^ H_2_O) throughout the experiment (Fig. 2D). On day 23, iWUE was similar across all plant lines. However, at the later days (particularly days 39 and 45), mean iWUE was highest in the overexpressors and lowest in the knockdowns, with WT showing an intermediate value. On day 45, mean iWUE was 25% higher in the overexpressors and 50% lower in the knockdowns, compared with WT (Fig. 2D).

Leaf 5 Chl *a* fluorescence analyses indicated that all plant lines exhibited similar ETR, NPQ and PSII excitation pressure (1−qP) on day 23, over a wide range of irradiance (see Supplementary Figs S6–S8). At later days, however, the overexpressors maintained higher ETR and lower NPQ and 1−qP than WT while the knockdowns maintained lower ETR and higher NPQ and 1−qP than WT, particularly at higher irradiance (Supplementary Figs S6–S8).

**Fig. 3.**
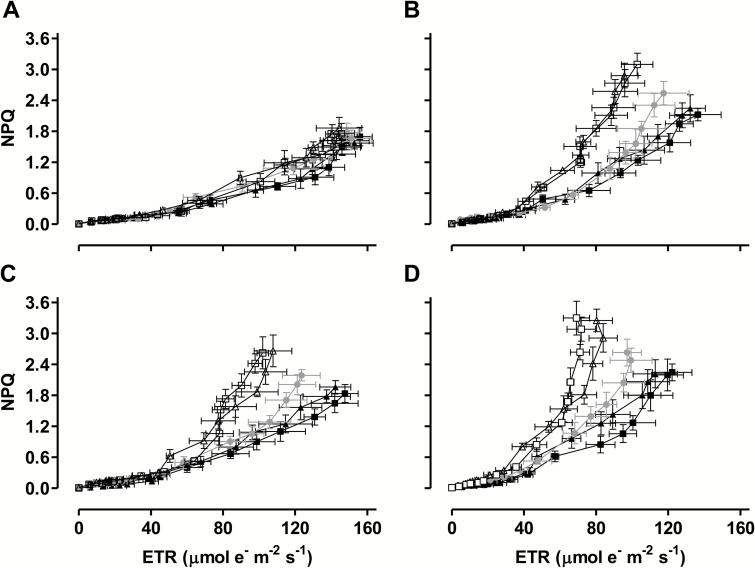
The relationship between ETR and NPQ at different times during a prolonged water deficit and measured at the growth irradiance. (A) Day 23. (B) Day 33. (C) Day 39. (D) Day 45. Data are the mean±SE of three independent experiments (*n*=3). Data are presented for WT (gray circle), AOX overexpressors (B7, closed triangle; B8, closed square), and AOX knockdowns (RI9, open triangle; RI29, open square). ETR and NPQ data for each individual line and over a wide range of measurement irradiances is presented in Supplementary Figs S6 and S7.

Figure 3 shows the relationship between NPQ and ETR. It indicates that, over the course of the experiment, the plants were able to generate a higher NPQ despite having a lower ETR. Further, this increased ‘sensitivity’ of NPQ to ETR was clearly enhanced in the knockdowns and reduced in the overexpressors, relative to WT, during the moderate water deficit period (days 33–45) (Fig. 3).

**Fig. 4.**
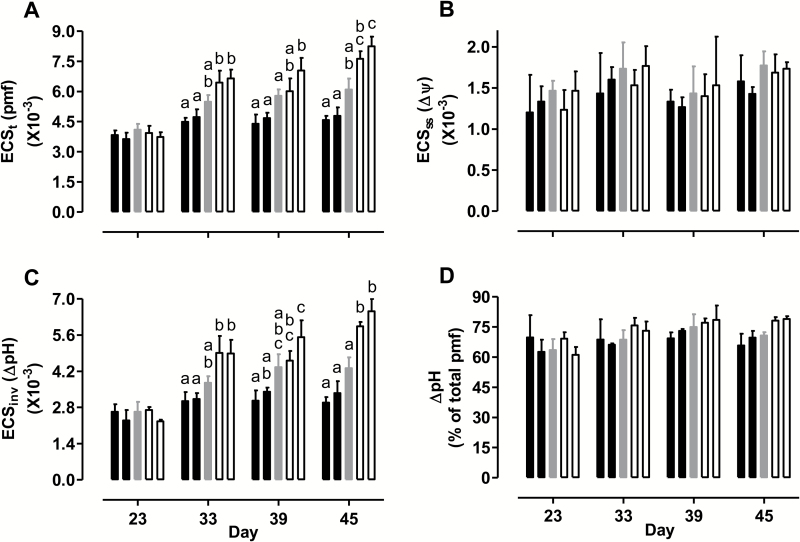
Thylakoid membrane pmf and the partitioning of pmf into its ∆ψ and ∆pH components in tobacco leaf at different times during a prolonged water deficit, and measured at the growth irradiance. (A) ECS_t_, a measure of pmf. (B) ECS_ss_, a measure of ∆ψ. (C) ECS_inv_, a measure of ∆pH. (D) ∆pH as a percentage of the total pmf. Data are shown for WT (gray bars), AOX overexpressors (B8, left closed bar; B7, right closed bar), and AOX knockdowns (RI9, left open bar; RI29, right open bar). Data are the mean±SE of three independent experiments (*n*=3). Within each data set, plant lines not sharing a common letter are significantly different from one another (*P*<0.05). In data sets without letters, there are no significant differences across plant lines.

Absorption spectroscopy combined with a DIRK analysis was used to both quantify leaf 5 pmf and partition the pmf into its ∆ψ and ∆pH components. On day 23, ECS_t_, a measure of pmf, did not differ across plant lines when measured at growth irradiance (Fig. 4A). However, by day 33, a pattern emerged where the overexpressors maintained a lower and the knockdowns maintained a higher ECS_t_ than WT. This pattern became more pronounced by day 39 and again by day 45. On day 45, ECS_t_ averaged 23% lower in the overexpressors and 30% higher in the knockdowns than in WT. These differences were not due to differences in the ∆ψ component of the pmf, measured as ECS_ss_, which remained similar across plant lines throughout the experiment (Fig. 4B). Instead, ∆pH (measured as ECS_inv_) differed across lines on days 33–45. For example, on day 45, mean ECS_inv_ was 26% lower in the overexpressors and 44% higher in the knockdowns, compared with WT (Fig. 4C). The percentage contribution of ∆pH toward the total pmf did not differ across plant lines (Fig. 4D).

**Fig. 5.**
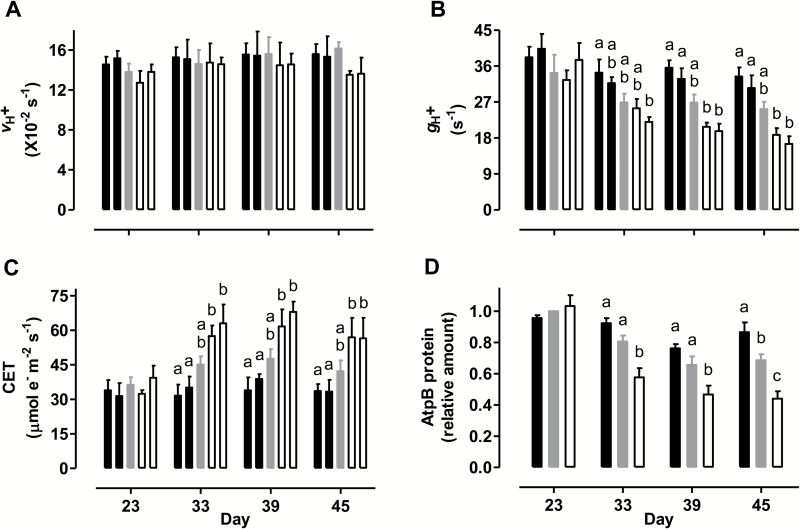
Thylakoid membrane proton flux-related parameters in tobacco leaf at different times during a prolonged water deficit. (A) The rate of proton flux from stroma to lumen. (B) The conductance of the thylakoid membrane for proton movement from lumen to stroma. (C) The rate of CET around PSI. (D) The amount of leaf AtpΒ protein. The measurements in (A–C) were made at the growth irradiance. In (A–C) data are presented for WT (gray bar), AOX overexpressors (B8, left closed bar; B7, right closed bar), and AOX knockdowns (RI9, left open bar; RI29, right open bar). In (D), data are presented for WT (gray bar), combined B8 and B7 (closed bar), and combined RI9 and RI29 (open bar). The AtpΒ protein amounts are relative to the amount in the WT on day 23, which was set to 1. All data are the mean±SE of three independent experiments (*n*=3). Within each data set, plant lines not sharing a common letter are significantly different from one another (*P*<0.05). In data sets without letters, there are no significant differences across plant lines.

The DIRK analysis was also used to quantify the rate of proton flux from stroma to lumen (*v*_H+_) and the conductance of the thylakoid membrane to proton movement from lumen to stroma (*g*_H+_) at the growth irradiance. In the WT, *v*_H+_ was relatively stable over the course of the experiment (Fig. 5A), while *g*_H+_ declined noticeably (by 22%) between day 23 and 33, and was then maintained at this lower conductance through day 45 (Fig. 5B). The *vH+* did not differ across plant lines on any of the days examined (Fig. 5A). On day 23, *g*_H+_ was also similar across plant lines. However, by day 33, a pattern emerged where the overexpressors maintained higher *g*_H+_ and the knockdowns maintained lower *g*_H+_ than WT. This pattern became more pronounced through days 39 and 45. On day 45, the mean *g*_H+_ was 23% higher in the overexpressors and 34% lower in the knockdowns than in WT (Fig. 5B). All of the above DIRK analyses were also performed at a saturating irradiance and yielded similar findings to those described above (Supplementary Figs S9 and S10).

Combined Chl fluorescence and PSI absorption spectroscopy analyses indicated that all plant lines exhibited similar rates of leaf 5 CET, measured at growth irradiance, on day 23 (Fig. 5C). At later days, however, the overexpressors maintained lower rates and the knockdowns maintained higher rates of CET than the WT. Supplementary Fig. S11 shows rates of CET of each plant line across a wide range of measurement irradiances.

**Fig. 6.**
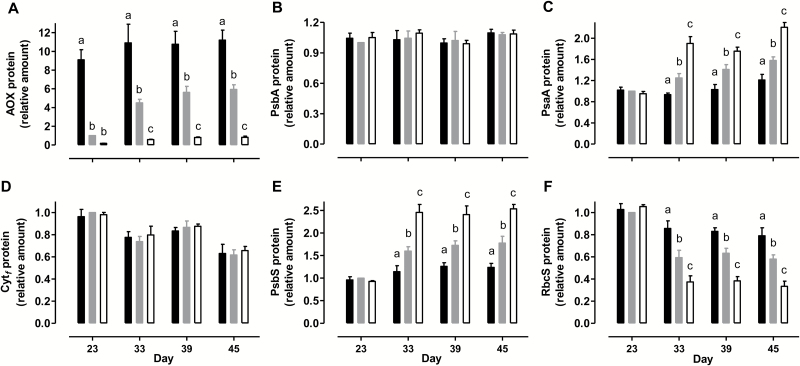
Amounts of AOX and different photosynthesis-related proteins in tobacco leaf at different times during a prolonged water deficit. (A) AOX. (B) PsbA. (C) PsaA. (D) Cyt_*f*_. (E) PsbS. (F) RbcS. Data are presented for WT (gray bar), combined B8 and B7 (closed bar), and combined RI9 and RI29 (open bar). Protein amounts are relative to the amount in the WT on day 23, which was set to 1. Data are the mean±SE of three independent experiments (*n*=3). Within each data set, plant lines not sharing a common letter are significantly different from one another (*P*<0.05). In data sets without letters, there are no significant differences across plant lines.

Immunoblot analyses compared the amount of AOX and several key photosynthetic proteins in leaf 5 across the plant lines. For these analyses, equal amounts of protein extract from RI9 and RI29 or B7 and B8 were combined prior to analysis. In WT, AOX protein amount increased strongly in response to moderate water deficit. Compared with day 23, AOX amount was increased 4.5-, 5.6-, and 5.9-fold on days 33, 39, and 45, respectively (Fig. 6A). This increase in AOX protein amount correlated with an increase in the excitation pressure being experienced by these plants (see Supplementary Fig. S12). We have reported before that growth excitation pressure appears an important factor influencing AOX amount across various growth conditions (Dahal *et al.*, 2017). As expected, AOX protein amount was always higher in the overexpressors and lower in the knockdowns, compared with WT. On day 23, AOX amount in overexpressors was 9.1-fold higher than WT, while AOX amount in knockdowns was only 17% of the WT amount. During moderate water deficit, AOX amount remained 2-fold higher in overexpressors than WT, while AOX amount in knockdowns was only 14% of the WT amount (average of days 33, 39, and 45) (Fig. 6A).

Of the six different photosynthetic proteins examined, none differed in amount between WT, overexpressors, and knockdowns during mild water deficit (day 23) (Figs 5D and 6). However, the amount of some proteins did differ dramatically across plant lines during the moderate water deficit period. In WT, the protein amount of AtpΒ (the Β-subunit of the F_1_ catalytic subcomplex of ATP synthase) was 28% lower during moderate water deficit (average of days 33, 39, and 45) compared with mild water deficit (Fig. 5D). However, this decline was less severe in the overexpressors and more severe in the knockdowns. Hence, the AtpΒ protein amount during moderate water deficit (average of days 33, 39, and 45) was 19% higher in the overexpressors and 31% lower in the knockdowns, compared with WT (Fig. 5D).

The protein amount of PsbA (D1 reaction center protein of PSII) was unchanged over the course of the experiment and did not differ across plant lines (Fig. 6B). However, PsaA (a reaction center protein of PSI) increased in response to moderate drought. Compared with day 23, PsaA amount in the WT was increased 1.3-, 1.4-, and 1.6-fold on days 33, 39, and 45, respectively. Further, this increase was exaggerated in the knockdowns but less evident in the overexpressors. Hence, the PsaA amount during moderate water deficit (average of days 33, 39, and 45) was 38% higher in the knockdowns and 25% lower in the overexpressors, compared with WT (Fig. 6C). Cyt_*f*_ (the *c*-type cyt subunit of the Cyt *b*_6_*f* complex) amount was more variable over the course of the experiment, but was lower during moderate than mild water deficit. However, the dynamic changes in Cyt_*f*_ amount were always similar across plant lines (Fig. 6D). In the WT, PsbS (a PSII-associated sensor of lumen pH necessary for NPQ induction) amount increased in response to moderate water deficit. Compared with day 23, PsbS amount was increased 1.6-, 1.7-, and 1.8-fold on days 33, 39, and 45, respectively. This increase was enhanced in the knockdowns, but less evident in the overexpressors. Hence, the PsbS amount during moderate water deficit (average of days 33, 39, and 45) was 45% higher in the knockdowns and 29% lower in the overexpressors, compared with WT (Fig. 6E). Compared with mild water deficit (day 23), RbcS (small subunit of Rubisco) amount in the WT declined by 40% during the moderate water deficit period (average of days 33, 39, and 45). This decline was more severe in the knockdowns and less severe in the overexpressors. Hence, the RbcS amount during moderate water deficit (average of days 33, 39, and 45) was 40% lower in the knockdowns and 37% higher in the overexpressors, compared with WT (Fig. 6F). For all of the immunoblot analyses described above, representative Western blots are shown in Supplementary Fig. S13.

### AOX respiration improves growth during a prolonged water deficit

**Fig. 7.**
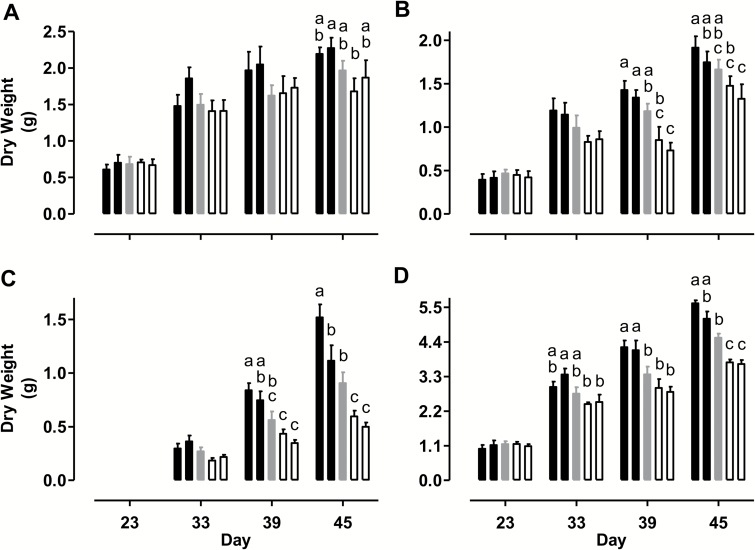
Tobacco plant DW at different times during a prolonged water deficit. Data are shown for (A) leaves, (B) stem, (C) reproductive tissue (flowers, seed pods) and (D) total shoot [sum of (A–C)]. Data are presented for WT (gray bar), AOX overexpressors (B8, left closed bar; B7, right closed bar), and AOX knockdowns (RI9, left open bar; RI29, right open bar). Data are the mean±SE of three independent experiments (*n*=3). Within each data set, plant lines not sharing a common letter are significantly different from one another (*P*<0.05). In data sets without letters, there are no significant differences across plant lines.

On day 23, all plant lines displayed similar leaf, stem and total above-ground biomass, measured as DW (Fig. 7). However, by day 45, total above-ground biomass averaged 19% higher in the AOX overexpressors and 18% lower in the AOX knockdowns, compared with WT (Fig. 7D). This difference across plant lines was primarily due to differences in the biomass of stem tissue and reproductive tissue (flowers and seed pods) (Fig. 7B, C). In these tissues, the biomass trends across plant lines mirrored the trends seen in leaf 5 *A* rates across the plant lines during the moderate water deficit period.

Reproductive tissue as a percentage of total above-ground biomass declined with declining AOX amount. On day 45, reproductive tissue averaged 24.5% of total biomass in the overexpressors, 20% of total biomass in the WT, and just 15% of total biomass in the knockdowns. On the other hand, total leaf biomass by day 45 (and on the other days) was more variable across plant lines, although there was still some tendency for elevated leaf biomass in the overexpressors (13% higher mean value than WT on day 45) and reduced leaf biomass in the knockdowns (10% lower mean value than WT on day 45) (Fig. 7A). There were also no clear differences in total leaf area across the plant lines on any days (see Supplementary Fig. S14). A separate set of experiments only evaluated plant DW (leaf, stem, and reproductive tissue) at day 45 and yielded similar results to all those described above (Supplementary Fig. S15). In this case, total above-ground biomass averaged 17% higher in the overexpressors and 27% lower in the knockdowns than in WT.

**Fig. 8.**
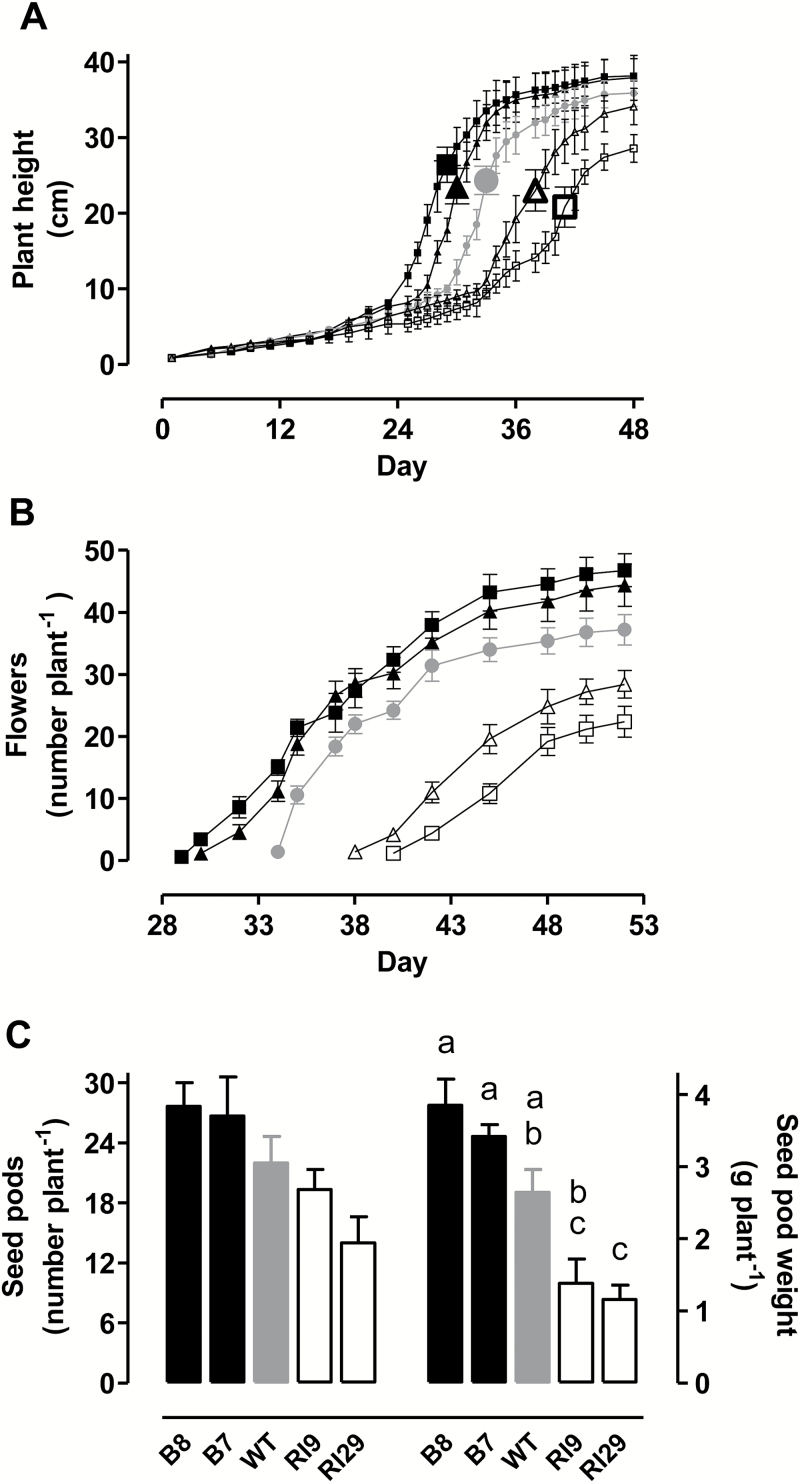
Tobacco growth and development during a prolonged water deficit. (A) Plant height over time. (B) Total number of flowers emerged over time. (C) Total number of seed pods (left axis and data set) and total DW of seed pods (right axis and data set). In (A, B) data are presented for WT (gray circle), AOX overexpressors (B7, closed triangle, B8, closed square), and AOX knockdowns (RI9, open triangle; RI29, open square), and are the mean±SE of three to five independent experiments (*n*=3–5). The large symbols in (A) denote the day when first flower(s) appeared. In (C) the data were collected following a water deficit experiment extended until day 92, when all plants were completely senesced and dry; and data are the mean±SE of three independent experiments (*n*=3). Within each data set, plant lines not sharing a common letter are significantly different from one another (*P*<0.05). In data sets without letters, there are no significant differences across plant lines.

Measurements of plant height indicated an earlier stem bolting in overexpressors and a delayed stem bolting in knockdowns, compared with WT (Fig. 8A). Further, the appearance of first flowers occurred 3–4 days earlier in the overexpressors and 5–8 days later in the knockdowns, compared with WT (Fig. 8B). Interestingly, though, plant height at the time of emergence of the first flowers (large data points in Fig. 8A) remained similar across the plant lines.

A separate set of experiments extended the moderate water deficit beyond day 45, to day 92. At day 92, the mean final number of seed pods per plant was 23% higher in the overexpressors and 24% lower in the knockdowns than in WT (Fig. 8C). Further, total seed pod weight was 38% higher in the overexpressors and 52% lower in the knockdowns than in WT (Fig. 8C). A germination test indicated that, regardless of plant line, the seed being generated was viable (i.e. >90% germination rate).

## Discussion

The metabolism and growth of WT tobacco were compared with those of AOX knockdown (RI9, RI29) and overexpression (B7, B8) lines. The comparison was of plants that had experienced a mild to moderate water deficit since the seedling stage, which will have allowed them to acclimate to the limited water availability (Harb *et al.*, 2010; Hummel *et al.*, 2010; Baerenfaller *et al.*, 2012; Schӧttler and Tόth, 2014; Avramova *et al.*, 2015; Clauw *et al.*, 2015; Bechtold *et al.*, 2016). A high AOX amount (overexpression lines) increased both photosynthesis and growth, while a low AOX amount (knockdown lines) compromised photosynthesis and growth, relative to WT plants with intermediate amounts of AOX. The differences in photosynthesis and growth across plant lines were not due to differences in leaf water relations or leaf expansion, both of which are key parameters to consider when examining plant performance, particularly under water deficit (Lawlor, 2013; Weraduwage *et al.*, 2015). For example, there were no obvious AOX-dependent differences across the plant lines in leaf RWC, *g*_s_, *T*, total leaf area, leaf size, total leaf DW, or specific leaf weight on any of the days examined. Instead, AOX amount appeared to primarily influence fundamental biochemical regulatory mechanisms in the chloroplast that are known to control and fine-tune photosynthetic activity in response to metabolic and environmental conditions. In turn, photosynthetic activity strongly influenced growth, particularly of reproductive sinks, despite the water limitation. These are the subjects of the discussion below.

### Maintenance of chloroplast energy balance by the mitochondrion is a critical factor preventing a biochemical limitation of photosynthesis induced by water deficit

We previously showed that when tobacco plants, having been grown and developed under well-watered conditions, were subjected to a relatively rapid onset of water deficit, leaf *R*_L_ was reprogrammed. There was a strong increase in AOX respiration alongside a strong decline in cyt oxidase capacity (Dahal *et al.*, 2014; Dahal and Vanlerberghe, 2017). Such a shift in the path of electron flow reduces the ATP yield of the mitochondrion, while maintaining its ability to act as an electron sink. Under these rapid-onset, short-term stress conditions, where *A* and hence ATP and NADPH consumption by the Calvin cycle were being strongly curtailed, this shift in mitochondrial metabolism supported chloroplast energy balance (Dahal and Vanlerberghe, 2017). The current study extends previous findings by showing that, even in plants where long-term growth and development has occurred under steady-state moderate water deficit conditions, the mitochondrion remains an essential player in supporting chloroplast energy balance. For example, excitation pressure, the fraction of closed (reduced) PSII reaction centers, was increased in the AOX knockdowns and reduced in the AOX overexpressors, compared with WT. This was evident despite long-term adjustments of the photosynthetic apparatus across plant lines that should counteract the excitation pressure differences. In particular, PSI content (measured as PsaA protein amount) was increased in the knockdowns and reduced in the overexpressors, compared with WT. All else being equal, this adjustment should alleviate excitation pressure in the knockdowns and elevate excitation pressure in the overexpressors, relative to WT. As discussed below, this impact of AOX on chloroplast energy balance during water deficit had significant effects on the paths of proton and electron flow (i.e. the proton circuit) in the thylakoid membrane, which then had important consequences for overall photosynthetic activity.

LET (or ETR) is coupled to proton influx from stroma to lumen. An important regulatory function of the resulting low lumen pH is the activation of NPQ, a key means to balance the synthesis of ATP and NADPH by the thylakoid reactions with the consumption of these intermediates by downstream metabolism, particularly the Calvin cycle (Foyer *et al.*, 2012; Tikhonov, 2013). Here, acclimation of WT tobacco to moderate water deficit was associated with a higher NPQ at any given ETR, as was also seen in wild watermelon (Kohzuma *et al.*, 2009). Such changes in the ‘sensitivity’ of NPQ to ETR is an important regulatory phenomenon since conditions that typically require a high NPQ, such as restrictions in downstream metabolism, are also conditions that will typically suppress ETR and the associated proton influx necessary to activate NPQ (Cruz *et al*, 2005; Armbruster *et al*, 2017). Avenson et al. (2004, 2005) emphasized four major mechanisms by which the sensitivity of NPQ to ETR might be increased. (i) While the H^+^/e^−^ ratio of LET is fixed, the overall H^+^/e^−^ ratio could be increased using CET. This supports further proton influx, and hence NPQ generation, without changing the rate of LET. (ii) Since the extent of the proton gradient established by LET also depends upon proton efflux from lumen to stroma, a decrease in the proton conductance of chloroplast ATP synthase is another potential means to enhance the sensitivity of NPQ to ETR. (iii) Since NPQ generation requires key protonation events, a change in the partitioning of the pmf away from ∆ψ and toward ∆pH could enhance the NPQ achieved by a given pmf. Changes in partitioning depend upon changes in the transport of counterions (Cruz *et al.*, 2001; Armbruster *et al.*, 2014; Herdean *et al.*, 2016). (iv) An increase in the capacity of components being controlled by protonation, such as PsbS or violaxanthin de-epoxidase, could enhance NPQ at a given proton concentration (Avenson *et al.*, 2004, 2005).

Interestingly, the increased sensitivity of NPQ to ETR during moderate water deficit was greater in the AOX knockdowns and less in the AOX overexpressors, compared with the WT response. This altered sensitivity across plant lines appeared to involve at least some of the mechanisms listed above. First, measured rates of CET were higher in the knockdowns and lower in the overexpressors, compared with WT. In fact, these differences in CET across plant lines may be underestimated since they depend upon the assumption that the partitioning of light energy absorption between PSII and PSI does not differ across the plant lines (Johnson, 2011). However, as discussed earlier, we found evidence that the PSI amount, but not the PSII amount, differed across the plant lines in response to moderate water deficit. Knockdowns had a higher amount of PsaA protein and overexpressors had a lower amount of PsaA protein than WT. If these differences across plant lines translate into similar differences in the partitioning of light energy absorption between the photosystems, then the actual differences in CET across lines will be greater than reported here. Regardless of actual rates, the qualitative differences in CET across plant lines provides an explanation of why rates of *vH+*, which is a measure of total proton influx regardless of it being supported by LET or CET, did not differ appreciably across the plant lines despite their differing ETR. The knockdowns are compensating for their lower ETR and associated proton influx, relative to WT, by increasing CET and its associated proton influx. Similarly, the overexpressors are compensating for their higher ETR by displaying lower rates of CET (Shikanai and Yamamoto, 2017).

Besides modulating CET, the difference in sensitivity of NPQ to ETR across plant lines was also achieved by manipulating proton efflux from lumen to stroma. The ECS analyses showed that, while moderate water deficit reduced *g*_H+_ in all plant lines compared with the mild water deficit condition, this reduction was most severe in the knockdowns and least severe in the overexpressors, with WT showing an intermediate response. Changes in *g*_H+_ can be achieved through long-term coarse control of ATP synthase protein amount and/or short-term fine biochemical control of ATP synthase activity (Kanazawa and Kramer, 2002; Kiirats *et al.*, 2009; Yamori *et al.*, 2011*b*; Schӧttler *et al.*, 2015; Carrillo *et al.*, 2016; Kohzuma *et al.*, 2017; Schmidt *et al.*, 2017). Here, WT tobacco responded to a prolonged moderate water deficit by reducing ATP synthase amount (estimated by AtpΒ protein amount) by 19–34%, depending on the day. The measured *g*_H+_ declined by a similar amount (22–27%), suggesting that coarse control of ATP synthase protein amount could largely explain the change in *g*_H+_. A similar conclusion can be drawn for the transgenic lines. In the knockdowns during moderate water deficit (average of three days and two lines), *g*_H+_ and AtpΒ were 41% and 52% lower, respectively, than under mild water deficit. On the other hand, *g*_H+_ and AtpB in the overexpressors during moderate water deficit were 16% and 11% lower, respectively, than under mild water deficit.

A change in the partitioning of the pmf away from ∆ψ and toward ∆pH could be another means to enhance NPQ at a given ETR. Here, ∆pH as a percentage of the total pmf increased slightly over the course of the experiment in all plant lines. Also, the knockdowns tended to display a slightly higher partitioning toward ∆pH, and the overexpressors tended to display a slightly lower partitioning toward ∆pH, compared with WT, during moderate water deficit. However, these differences were relatively minor compared with the differences in CET and ATP synthase amount described above. A larger difference was seen in the protein amount of PsbS across plant lines. Hence, it could also be contributing toward the differences in sensitivity of NPQ to ETR across the plant lines.

A consensus has developed that declines in photosynthesis during water deficit can be the result of biochemical limitation(s) that reduce *A* over and above that due to stomatal closure and the ensuing CO_2_ limitation (Lawlor, 2002; Flexas *et al.*, 2004; Grassi and Magnani, 2005; Lawlor and Tezara, 2009; Rivero *et al.*, 2009; Pinheiro and Chaves, 2011; Perez-Martin *et al.*, 2014; Zhou *et al.*, 2014). In several species, this biochemical limitation has been linked to ATP synthase amount and activity, suggesting this to be a widespread phenomenon (Tezara *et al.*, 1999; Kohzuma *et al.*, 2009; Hoshiyasu *et al.*, 2013; Dahal *et al.*, 2014). Modulation of ATP synthase acts to define the upper limit of ETR (and hence *A*) that is possible before becoming constrained by rising NPQ, as well as increased photosynthetic control at the cyt *b*_6_*f* complex (Rott *et al.*, 2011; Foyer *et al.*, 2012; Tikhonov, 2014). Based on the current study, it is clear that AOX respiration impacts chloroplast energy balance during water deficit, disrupting the thylakoid proton circuit, and hence influencing the onset of this biochemical limitation of photosynthesis.

Given the above discussion, a critical question is, what is the signal(s) controlling ATP synthase amount? In other words, what metabolic conditions under water deficit are triggering the ATP synthase decline? This decline was less severe in AOX overexpressors and more severe in AOX knockdowns, compared with WT, which might provide a clue(s) to the metabolic conditions responsible. Under water deficit, AOX amount is a strong determinant of *R*_L_, which in turn correlates strongly with excitation pressure (Dahal and Vanlerberghe, 2017; Dahal *et al.*, 2017). When AOX is lower, *R*_L_ is lower and excitation pressure is higher. This is strong evidence that consumption of electrons by AOX is important in preventing an over-reduction of the photosynthetic electron transport chain during water deficit. Such over-reduction, or some consequence of this over-reduction (e.g. oxidative damage or ROS signaling; Mahler *et al.*, 2007, Lawlor and Tezara, 2009; Noctor *et al.*, 2014; Foyer *et al.*, 2017) might be the condition responsible for down-regulation of ATP synthase amount. While the cyt pathway can also consume electrons, a concerted shift away from this path and toward AOX will lower the mitochondrial ATP yield per electron consumed. This might allow increased activity of additional chloroplast electron sinks (e.g. Mehler reaction) otherwise restricted by rates of ATP turnover (Dahal *et al.*, 2015). Hence, some component of adenylate energy status, such as ATP or P_i_ amount is another potential underlying factor that could be controlling ATP synthase amount (Shikanai and Yamamoto, 2017).

Previous studies have noted that water deficit-induced biochemical limitations of photosynthesis can coincide with declines in Rubisco amount or activity (Tezara *et al.*, 2002; Dahal *et al.*, 2014). Here, the changes in AtpΒ and RbcS amount were remarkably similar, suggesting that their amounts are being tightly coordinated. This is perhaps part of a more general system acting to harmonize the capacity of the thylakoid and stromal reactions (Yamori *et al.*, 2010; Schӧttler and Tόth, 2014; Yang *et al.*, 2016*a*). Hence, our current hypothesis is that the change in Rubisco amount is a secondary consequence of the change in ATP synthase amount.

### Biochemical limitations of photosynthesis can have a significant impact on water use efficiency and growth during water deficit

A potentially powerful approach to improve plant performance under water-limiting conditions is to increase iWUE, the ratio of instantaneous CO_2_ uptake to water loss by the leaf during photosynthesis (Araus *et al.*, 2002; Condon *et al.*, 2002, 2004; Polley, 2002; Gago *et al.*, 2014). This could be achieved by decreasing *T*, increasing *A*, or by some favorable combination of the two. In biotechnological studies in which leaf iWUE has been shown to differ across plant lines, improved iWUE has almost always been associated with a decrease in *T*, due, for example, to changes in stomatal density or function (reviewed in Lawlor, 2013). In other studies, the efficiency of whole plant water use has been made more favorable, due, for example, to broader changes in leaf and/or root growth and development (Masle *et al.*, 2005; Karaba *et al.*, 2007; Thompson *et al.*, 2007; Yu *et al.*, 2008; Rivero *et al.*, 2009; Lawlor, 2013; Yang *et al.*, 2016*b*; Hughes *et al.*, 2017). Nonetheless, using the above approaches to increase plant performance and yield has proven challenging since, while they may improve performance during water deficit, they are also often associated with a growth penalty when water is not limiting (Lawlor, 2013). When raised under well-watered conditions or when experiencing only a mild water deficit, the WT, knockdown and overexpression plants used here all display similar *A* and *T* under a wide range of growth and measurement irradiances (Dahal *et al.*, 2017). However, the current study indicates that, once a threshold severity of prolonged water deficit is achieved, large differences in iWUE develop across the plant lines, due solely to differences in *A*. This demonstrates that, through a manipulation in photosynthesis–respiration interactions, it is possible to increase iWUE, photosynthesis and growth under water-limiting conditions, and without any obvious compromising of performance when water is more plentiful (Nunes-Nesi *et al.*, 2011).

One consequence of the growth conditions used in this study was that the plants were still relatively small when they began to flower. This likely represents ‘stress-induced flowering’, a developmental phenomenon documented in many species and in response to diverse abiotic stresses, including water deficit (Takeno, 2016). Further, there were clear differences in flowering time across the plant lines, with overexpressors flowering earlier and knockdowns flowering later. Interestingly, when Liu *et al.* (2009) compared WT tobacco to transgenic lines with increased or decreased amount of a mitochondrial external NADPH dehydrogenase, they found that differences in plant height and flowering time across the plant lines correlated with differences in the NADPH/NADP^+^ ratio of specifically the stem tissue. High stem NADPH/NADP^+^ slowed increases in plant height and delayed flowering time. All plant lines did, however, flower once reaching a similar threshold height, which is similar to the findings here. The differences in flowering time across plant lines in the current study might represent an interesting system to elucidate cue(s) responsible for stress-induced flowering, which are poorly understood (Takeno, 2016).

Most of the final difference in shoot biomass accumulation across plant lines was due to the reproductive sinks (flowers, seeds). This indicates that biotechnological manipulations altering *A* may preferentially impact on these strong sinks for photosynthate, particularly under ‘stress-induced flowering’ conditions such as prolonged water deficit. Finally, our results may relate to a study in which AOX overexpression improved the growth of Arabidopsis seedlings exposed to osmotic stress or water deficit (Skirycz *et al.*, 2010). Whether photosynthesis played a role in those growth differences has, to our knowledge, not been reported.

## Supplementary data

Supplementary data are available at *JXB* online.

Fig. S1. The irrigation schedule used to compare the metabolism and growth of WT and transgenic tobacco plants during a prolonged water deficit.

Fig. S2. Water status of tobacco leaf at different times during a prolonged water deficit.

Fig. S3. Characteristics of tobacco leaf at different times during a prolonged water deficit.

Fig. S4. Tobacco leaf dimensions at different times during a prolonged water deficit.

Fig. S5. Tobacco leaf *A* rates as a function of irradiance (light response curves) during a prolonged water deficit.

Fig. S6. Tobacco leaf PSII ETR (LET) as a function of irradiance during a prolonged water deficit.

Fig. S7. Tobacco leaf NPQ as a function of irradiance during a prolonged water deficit.

Fig. S8. Tobacco leaf PSII excitation pressure as a function of irradiance during a prolonged water deficit.

Fig. S9. Thylakoid membrane pmf and the partitioning of pmf into its Δψ and ΔpH components in tobacco leaf at different times during a prolonged water deficit.

Fig. S10. Thylakoid membrane proton flux-related parameters in tobacco leaf at different times during a prolonged water deficit.

Fig. S11. Rates of CET around PSI in tobacco leaf as a function of irradiance and at different times during a prolonged water deficit.

Fig. S12. The relationship between PSII excitation pressure (1−qP) measured at the growth irradiance and leaf AOX protein amount in WT tobacco at different times during a prolonged water deficit.

Fig. S13. Representative immunoblots for AOX and several photosynthesis-related proteins at different times during a prolonged water deficit.

Fig. S14. Total tobacco plant leaf area at different times during a prolonged water deficit.

Fig. S15. Tobacco plant DW after a prolonged water deficit.

Supplementary Figures S1-S15

## Author contributions

KD performed most of the experiments and commented on the writing; KD and GCV designed the experiments and analysed the data; GCV conceived the project and wrote the article.

## Abbreviations:

*A*: net CO_2_ assimilation rate
AOX: alternative oxidase
CET: cyclic electron transport
cyt: cytochrome
DIRK: dark-interval relaxation kinetics
DW: dry weight
ECS: electrochromic shift
ETR: rate of linear electron transport through photosystem II
*g*_H+_: conductance of the thylakoid membrane to proton movement from lumen to stroma
*g*_s_: stomatal conductance to CO_2_
iWUE: instantaneous leaf water use efficiency
LET: linear electron transport
NPQ: non-photochemical-energy quenching
∆pH: chemical potential
∆ψ: electrical potential
PPFD: photosynthetic photon flux density
PSI: photosystem I
PSII: photosystem II
pmf: proton motive force
*R*_D_: rate of mitochondrial respiration in the dark
*R*_L_: rate of mitochondrial respiration in the light
RWC: relative water content
qP: photochemical energy quenching
*T*: transpiration rate
*v*_H+_: relative rate of proton flux from stroma to lumen
WT: wild-type.
