# Supplementary material for: Improved chloroplast energy balance during water deficit enhances plant growth: more crop per drop
Source: J Exp Bot. 2017 Dec 21;69(5):1183–97. doi: 10.1093/jxb/erx474 (PMC6018952; doi:10.1093/jxb/erx474)
Supplement: Supplementary Figures S1-S15 [file erx474_suppl_supplementary-figures-s1-s15.pdf]

Supplementary Figures S1-S15

Keshav Dahal and Greg C. Vanlerberghe

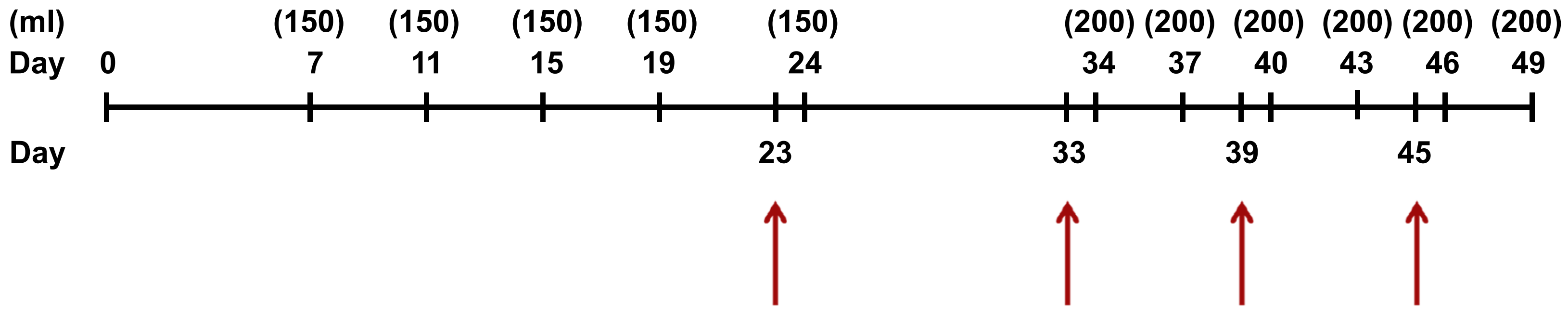

Supplementary Figure S1

Supplementary Figure S1 | The irrigation schedule used to compare the metabolism and growth of WT and transgenic tobacco plants during a prolonged water deficit. On day 0, the seedlings were transplanted to large pots (five seedlings per pot, one of each plant line) with soil saturated to field capacity, and transferred to a growth chamber. On days 7, 11, 15, 19 and 24, the pots were irrigated with 150 ml (30 ml/plant) of 1/10th strength Hoagland's solution. On days 34, 37, 40, 43, 46 and 49, the pots were irrigated with 200 ml (40 ml/plant) of 1/10th strength Hoagland's solution. Most physiological, biochemical and growth analyses were performed on day's 23, 33, 39 and 45 (red arrows). See Materials and Methods for further details regarding the growth conditions.

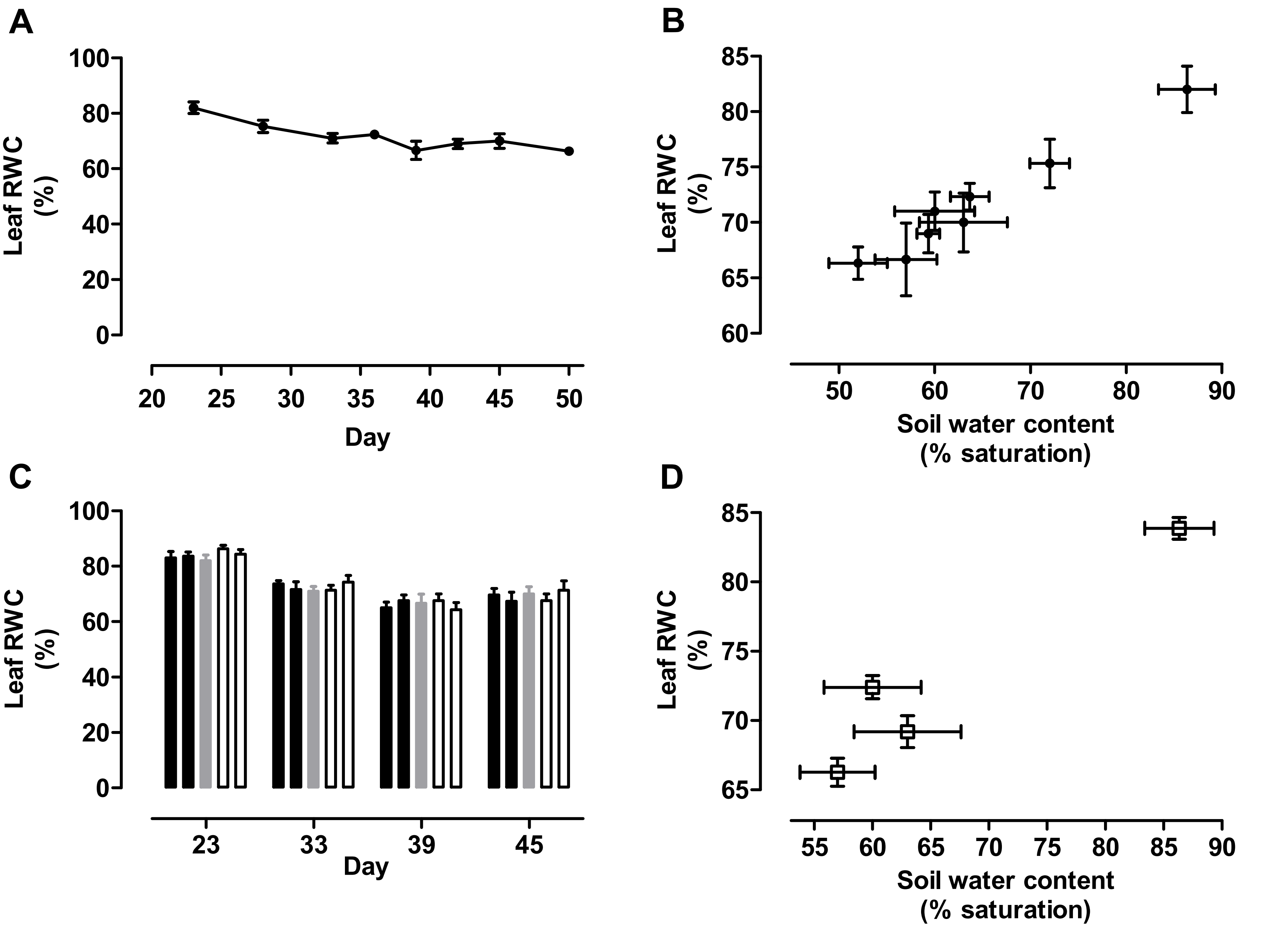

Supplementary Figure S2

Supplementary Figure S2 │ Water status of tobacco leaf at different times during a prolonged water deficit. (A) Leaf RWC of WT tobacco plants grown for 50 days in a growth chamber. (B) The relationship between soil water content and leaf RWC of WT tobacco plants grown for 50 days in a growth chamber. (C) Leaf RWC of tobacco plants grown for up to 45 days in a growth chamber. Data are shown for WT (grey bars), AOX overexpressors (B8, left closed bar, B7, right closed bar) and AOX knockdowns (RI9, left open bar; RI29, right open bar). (D) The relationship between soil water content and leaf RWC (average of all five plant lines) of tobacco plants grown for 45 days in a growth chamber, with measurements taken at day's 23, 33, 39 and 45. In all cases, the plants were grown under water deficit conditions using the irrigation schedule outlined in Supplementary Fig. S1. All data are the mean $\pm$ SE of three independent experiments (n=3). See Materials and Methods for further details regarding the growth conditions.

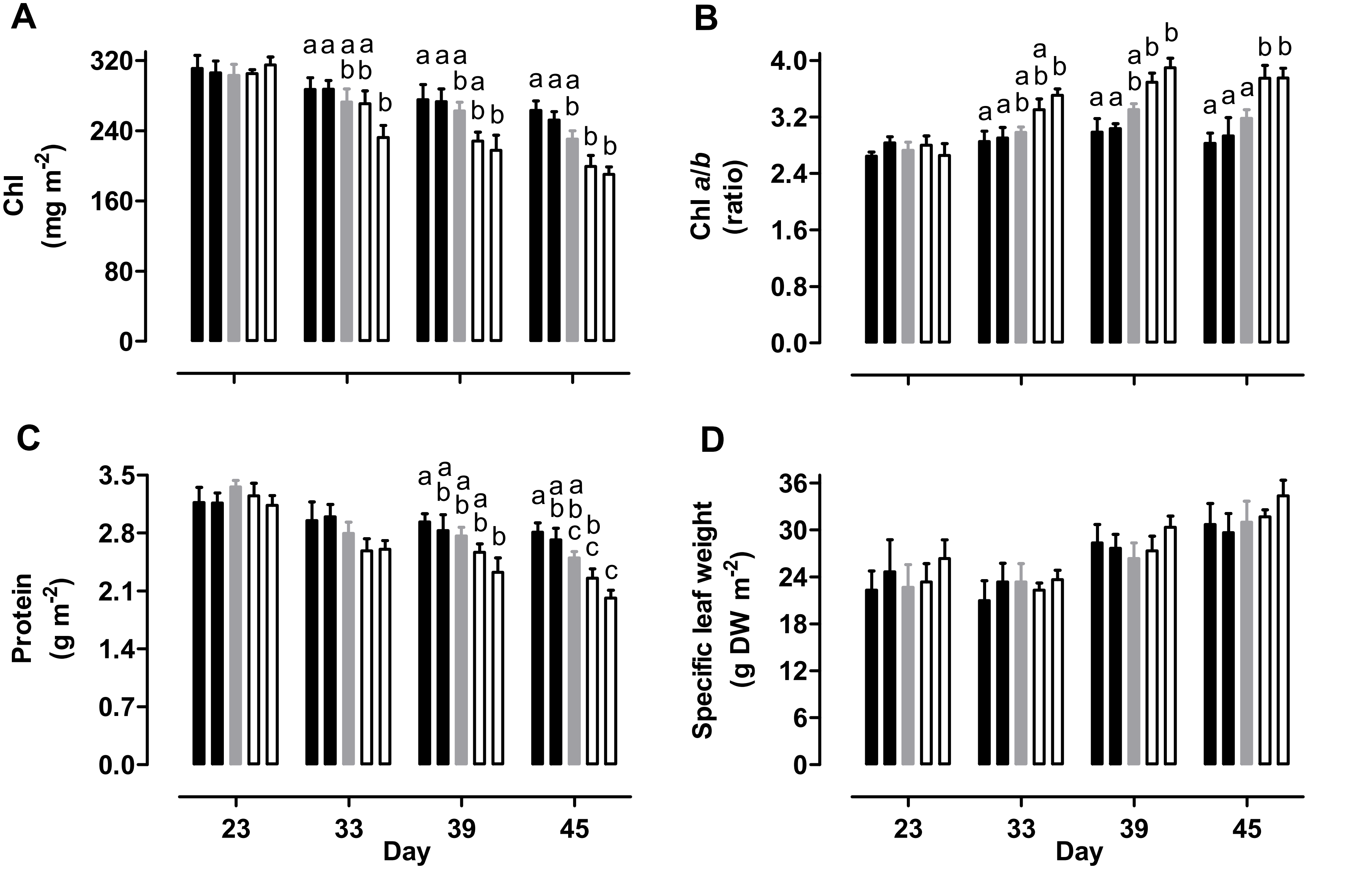

Supplementary Figure S3

Supplementary Figure S3 │ Characteristics of tobacco leaf at different times during a prolonged water deficit. (A) Total Chl. (B) Chl *a/b* ratio. (C) Total protein. (D) Specific leaf weight. Data are shown for WT (gray bars), AOX overexpressors (B8, left closed bar; B7, right closed bar) and AOX knockdowns (RI9, left open bar; RI29, right open bar). Data are the mean ± SE of three independent experiments (n=3). Within each data set, plant lines not sharing a common letter are significantly different from one another (P<0.05). In data sets without letters, there are no significant differences across plant lines.

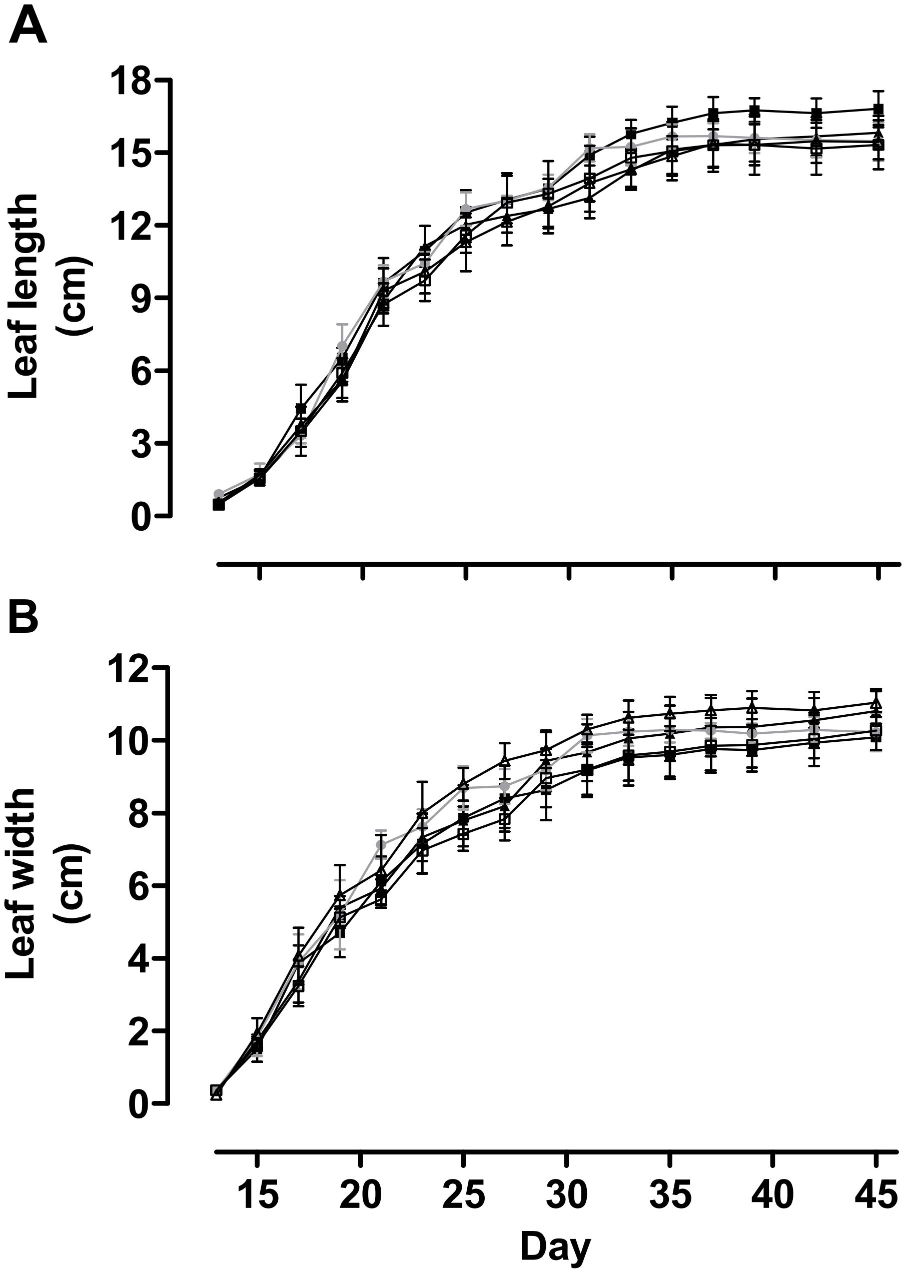

Supplementary Figure S4

Supplementary Figure S4 │ Tobacco leaf dimensions at different times during a prolonged water deficit. (A) Leaf 5 length. (B) Leaf 5 width. Data are presented for WT (grey circle), AOX overexpressors (B7, closed triangle; B8, closed square) and AOX knockdowns (RI9, open triangle; RI29, open square) and represent the mean ± SE of three independent experiments (n=3).

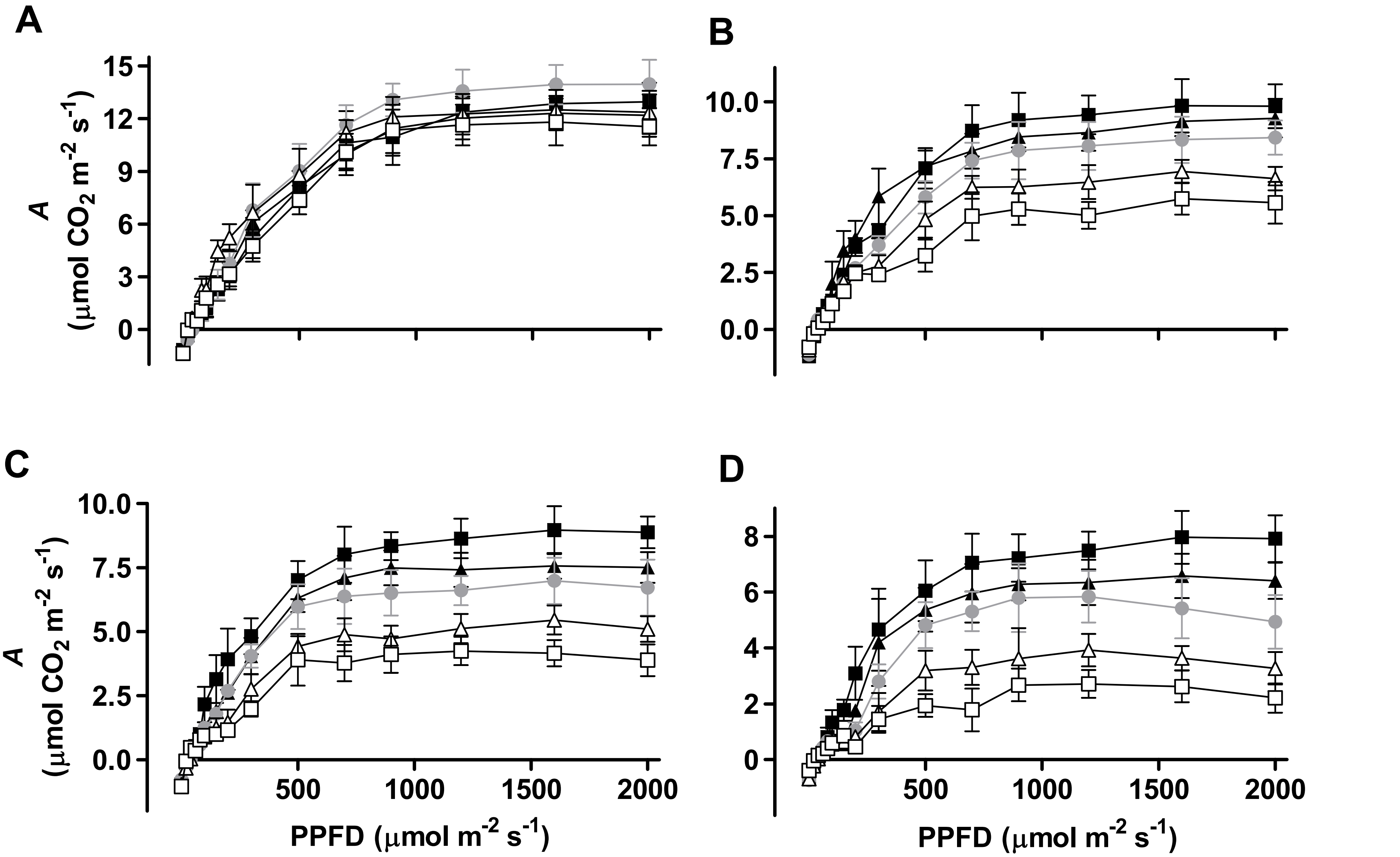

Supplementary Figure S5

Supplementary Figure S5 │ Tobacco leaf *A* rates as a function of irradiance (light response curves) during a prolonged water deficit. (A) Day 23. (B) Day 33. (C) Day 39. (D) Day 45. Data are presented for WT (grey circle), AOX overexpressors (B7, closed triangle; B8, closed square) and AOX knockdowns (RI9, open triangle; RI29, open square) and represent the mean $\pm$ SE of three independent experiments (n=3).

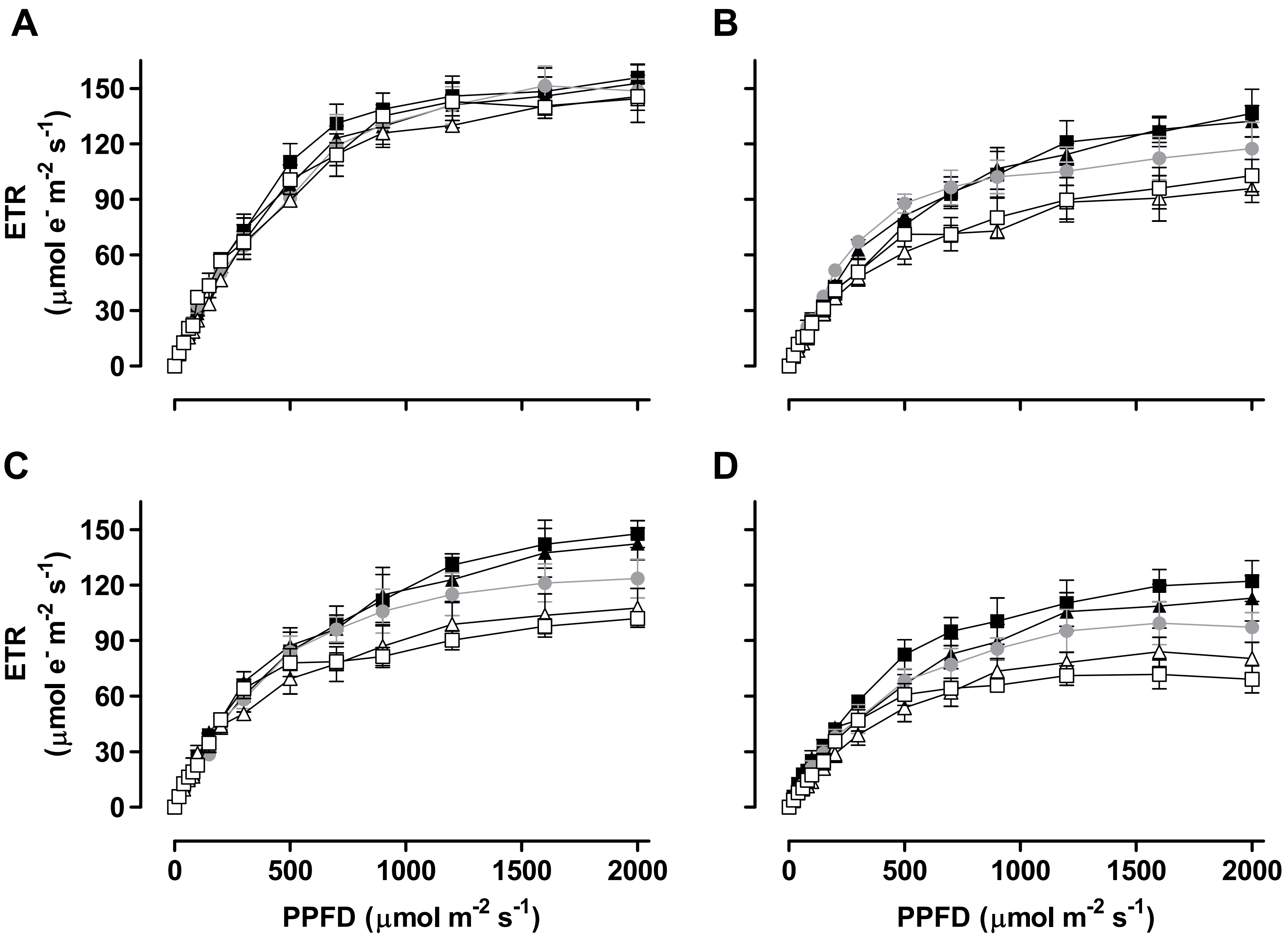

Supplementary Figure S6

Supplementary Figure S6 │ Tobacco leaf PSII ETR (LET) as a function of irradiance during a prolonged water deficit. (A) Day 23. (B) Day 33. (C) Day 39. (D) Day 45. Measurements were done alongside the light response curves of *A* shown in Supplementary Fig. S5. Data are presented for WT (grey circle), AOX overexpressors (B7, closed triangle; B8, closed square) and AOX knockdowns (RI9, open triangle; RI29, open square) and represent the mean ± SE of three independent experiments (n=3).

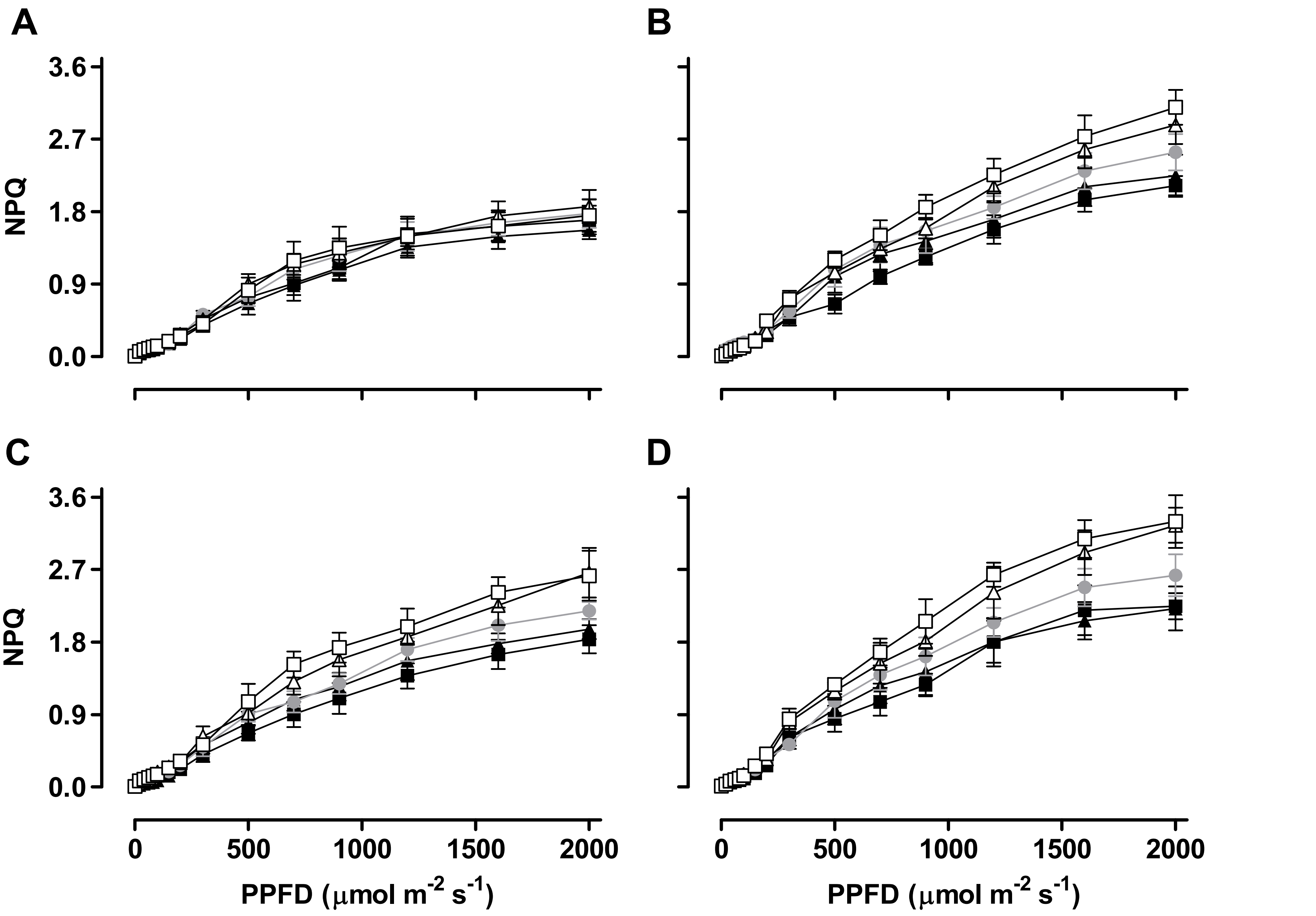

Supplementary Figure S7

Supplementary Figure S7 | Tobacco leaf NPQ as a function of irradiance during a prolonged water deficit. (A) Day 23. (B) Day 33. (C) Day 39. (D) Day 45. Measurements were done alongside the light response curves of *A* shown in Supplementary Fig. S5. Data are presented for WT (grey circle), AOX overexpressors (B7, closed triangle; B8, closed square) and AOX knockdowns (RI9, open triangle; RI29, open square) and represent the mean ± SE of three independent experiments (n=3).

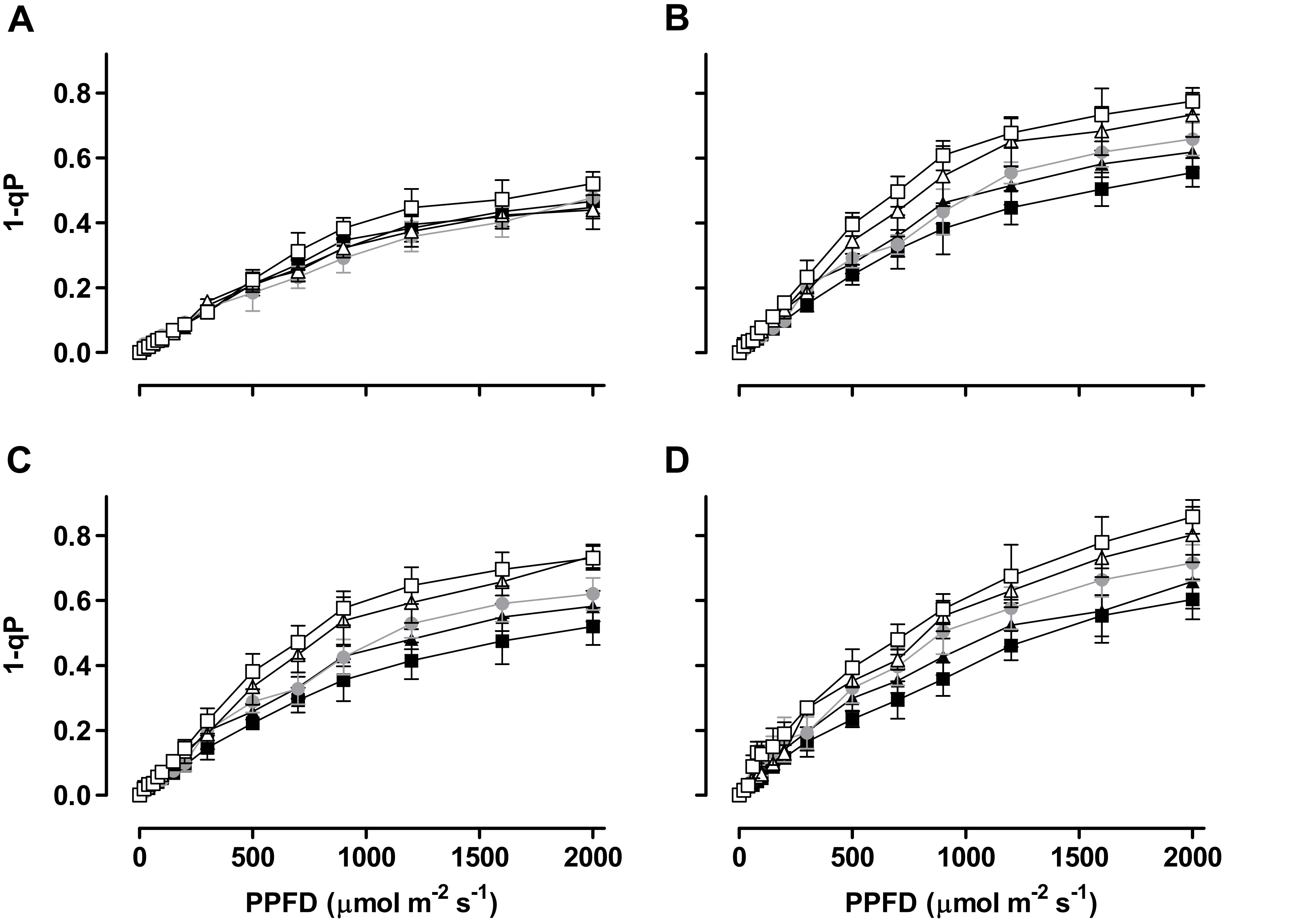

Supplementary Figure S8

Supplementary Figure S8 │ Tobacco leaf PSII excitation pressure as a function of irradiance during a prolonged water deficit. (A) Day 23. (B) Day 33. (C) Day 39. (D) Day 45. Measurements were done alongside the light response curves of *A* shown in Supplementary Fig. S5. Data are presented for WT (grey circle), AOX overexpressors (B7, closed triangle; B8, closed square) and AOX knockdowns (RI9, open triangle; RI29, open square) and represent the mean ± SE of three independent experiments (n=3).

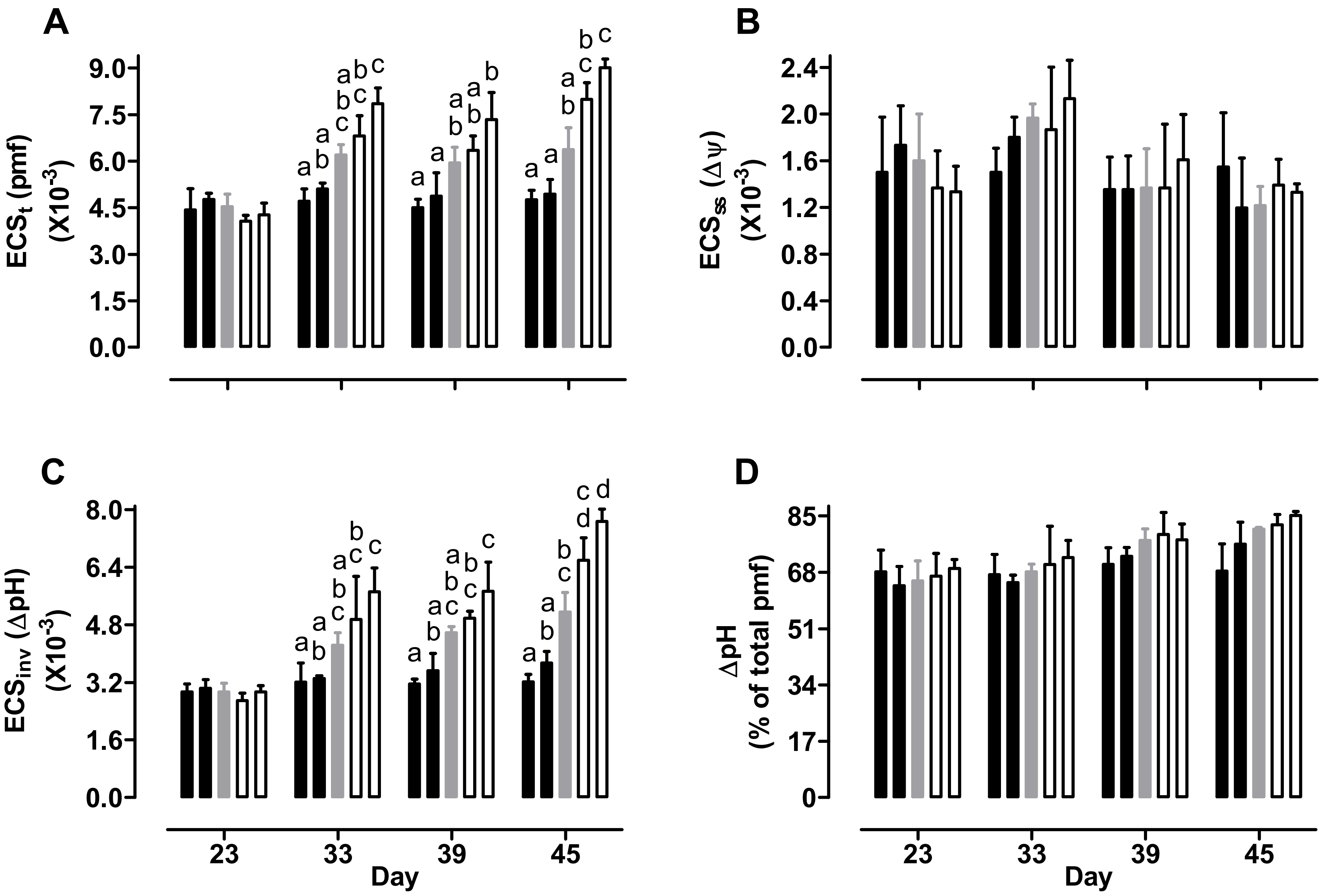

Supplementary Figure S9

Supplementary Figure S9 │ Thylakoid membrane pmf and the partitioning of pmf into its $\Delta\psi$ and ΔpH components in tobacco leaf at different times during a prolonged water deficit. (A) ECS_t, a measure of pmf. (B) ECS_{ss}, a measure of $\Delta\psi$. (C) ECS_{inv}, a measure of ΔpH. (D) ΔpH as a % of the total pmf. All measurements were done at 1600 PPFD. Data are presented for WT (grey bar), AOX overexpressors (B8, left closed bar; B7, right closed bar) and AOX knockdowns (RI9, left open bar; RI29, right open bar) and represent the mean $\pm$ SE of three independent experiments (n=3). Within each data set, plant lines not sharing a common letter are significantly different from one another ($P<0.05$). In data sets without letters, there are no significant differences across plant lines.

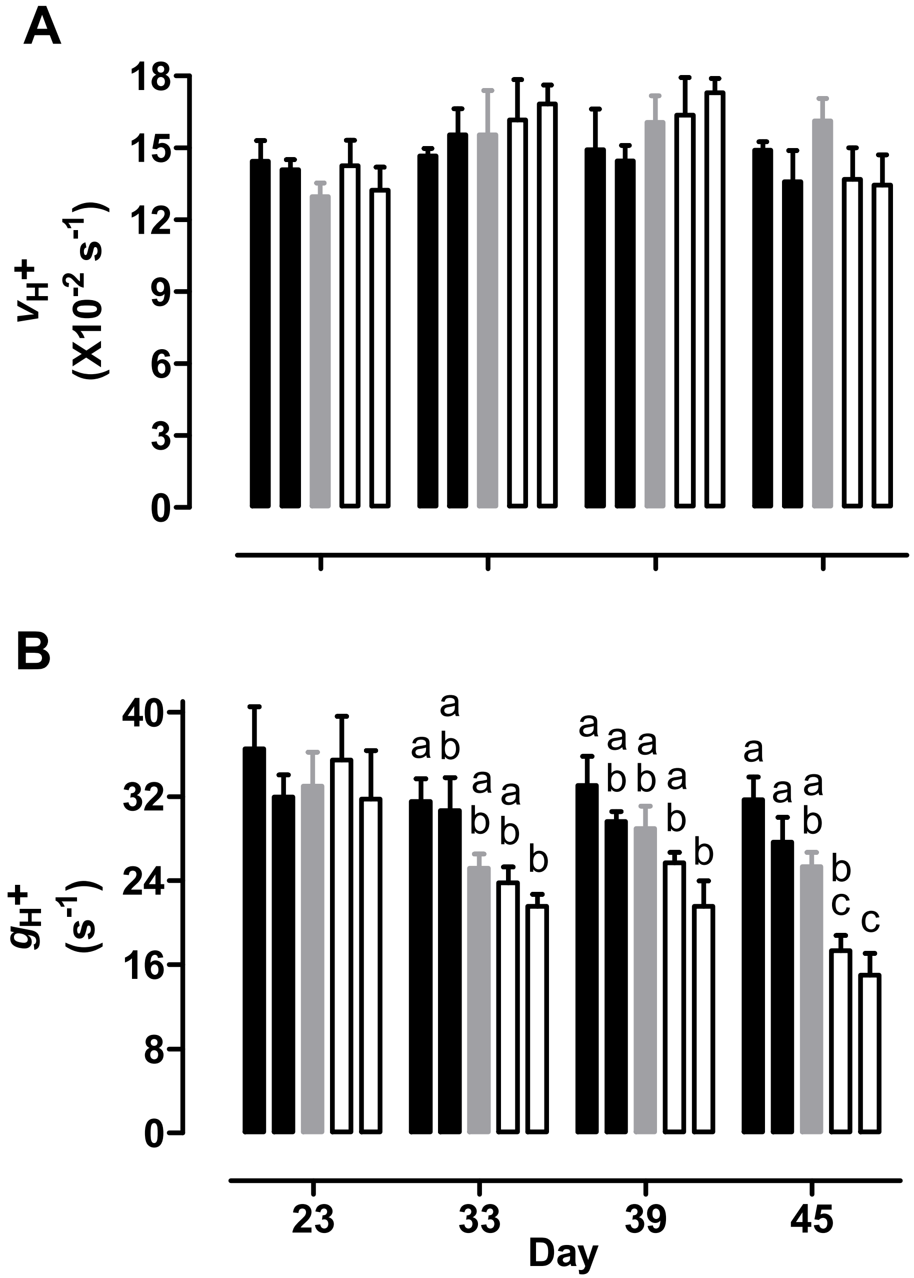

Supplementary Figure S10

Supplementary Figure S10 │ Thylakoid membrane proton flux-related parameters in tobacco leaf at different times during a prolonged water deficit. (A) The rate of proton flux from stroma to lumen. (B) The conductance of the thylakoid membrane for proton movement from lumen to stroma. All measurements were done at 1600 PPFD. Data are presented for WT (grey bar), AOX overexpressors (B8, left closed bar; B7, right closed bar) and AOX knockdowns (RI9, left open bar; RI29, right open bar) and represent the mean $\pm$ SE of three independent experiments (n=3). Within each data set, plant lines not sharing a common letter are significantly different from one another ($P<0.05$). In data sets without letters, there are no significant differences across plant lines.

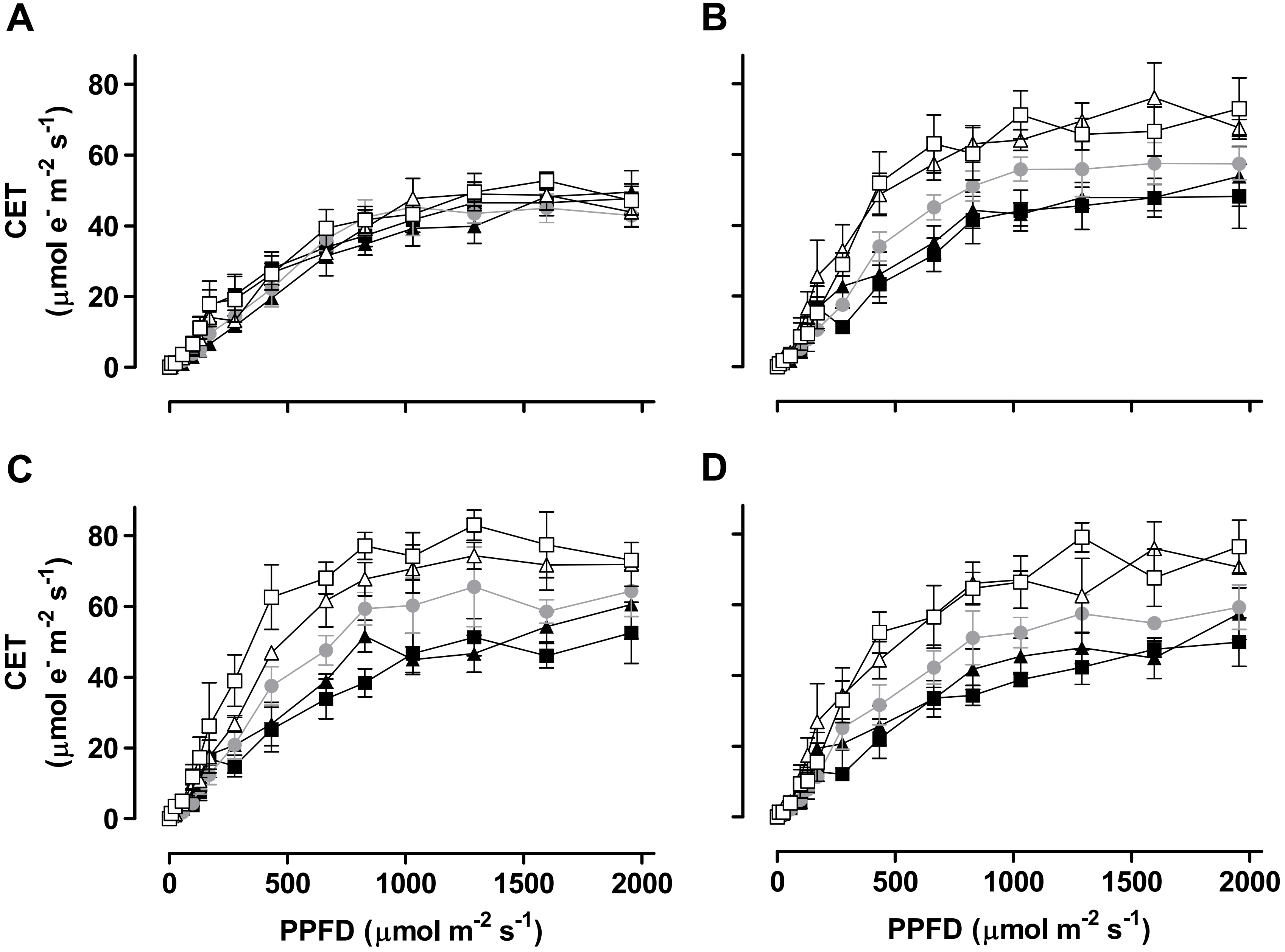

Supplementary Figure S11

Supplementary Figure S11 │ Rates of CET around PSI in tobacco leaf as a function of irradiance and at different times during a prolonged water deficit. (A) Day 23. (B) Day 33. (C) Day 39. (D) Day 45. Data are presented for WT (grey circle), AOX overexpressors (B7, closed triangle; B8, closed square) and AOX knockdowns (RI9, open triangle; RI29, open square) and represent the mean $\pm$ SE of three independent experiments (n=3).

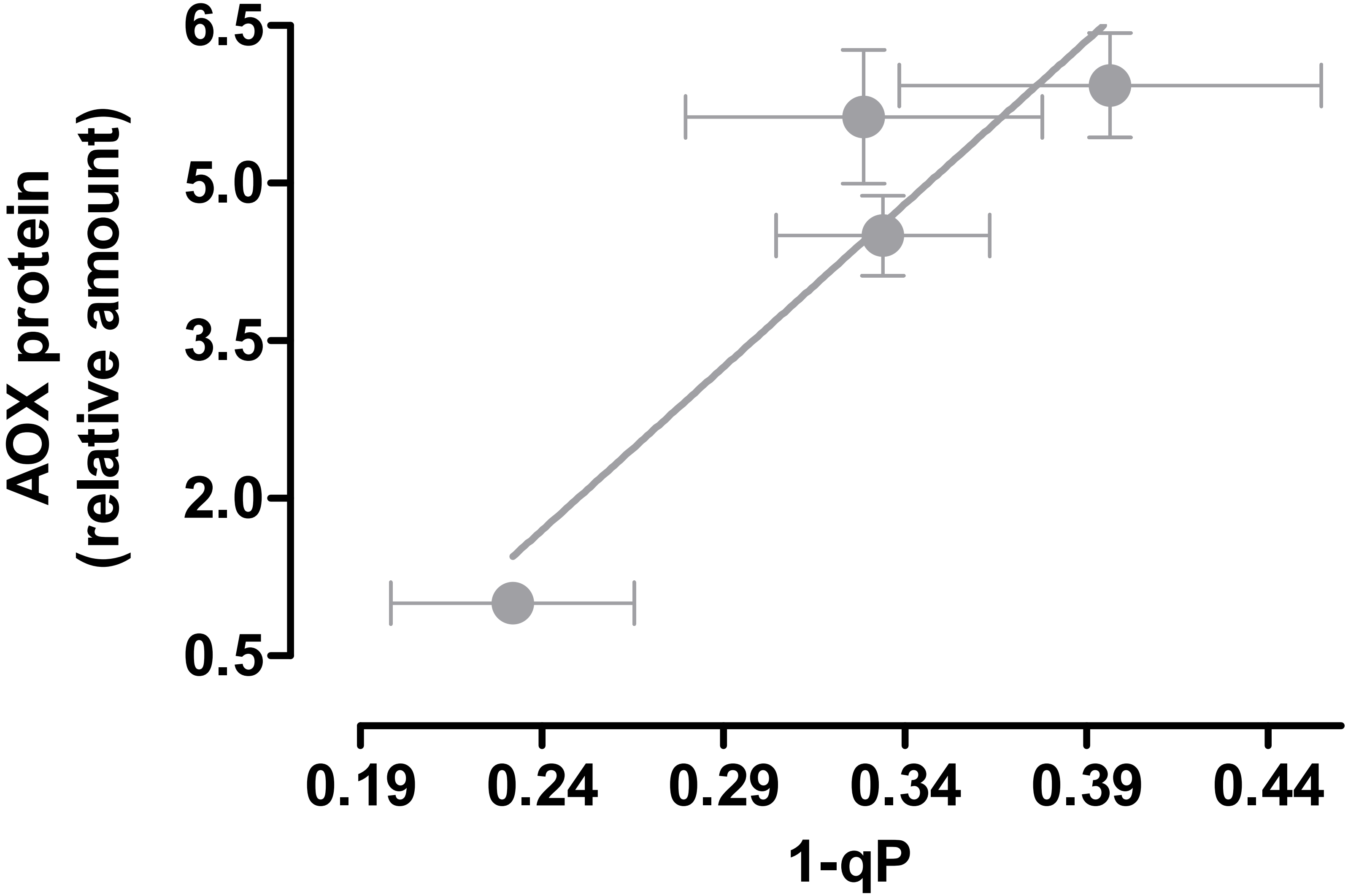

Supplementary Figure S12

Supplementary Figure S12 │ The relationship between PSII excitation pressure (1-qP) measured at the growth irradiance and leaf AOX protein amount in WT tobacco at different times during a prolonged water deficit. Data were measured at day's 23, 33, 39 and 45, and are the mean +/- S.E. of three independent experiments.

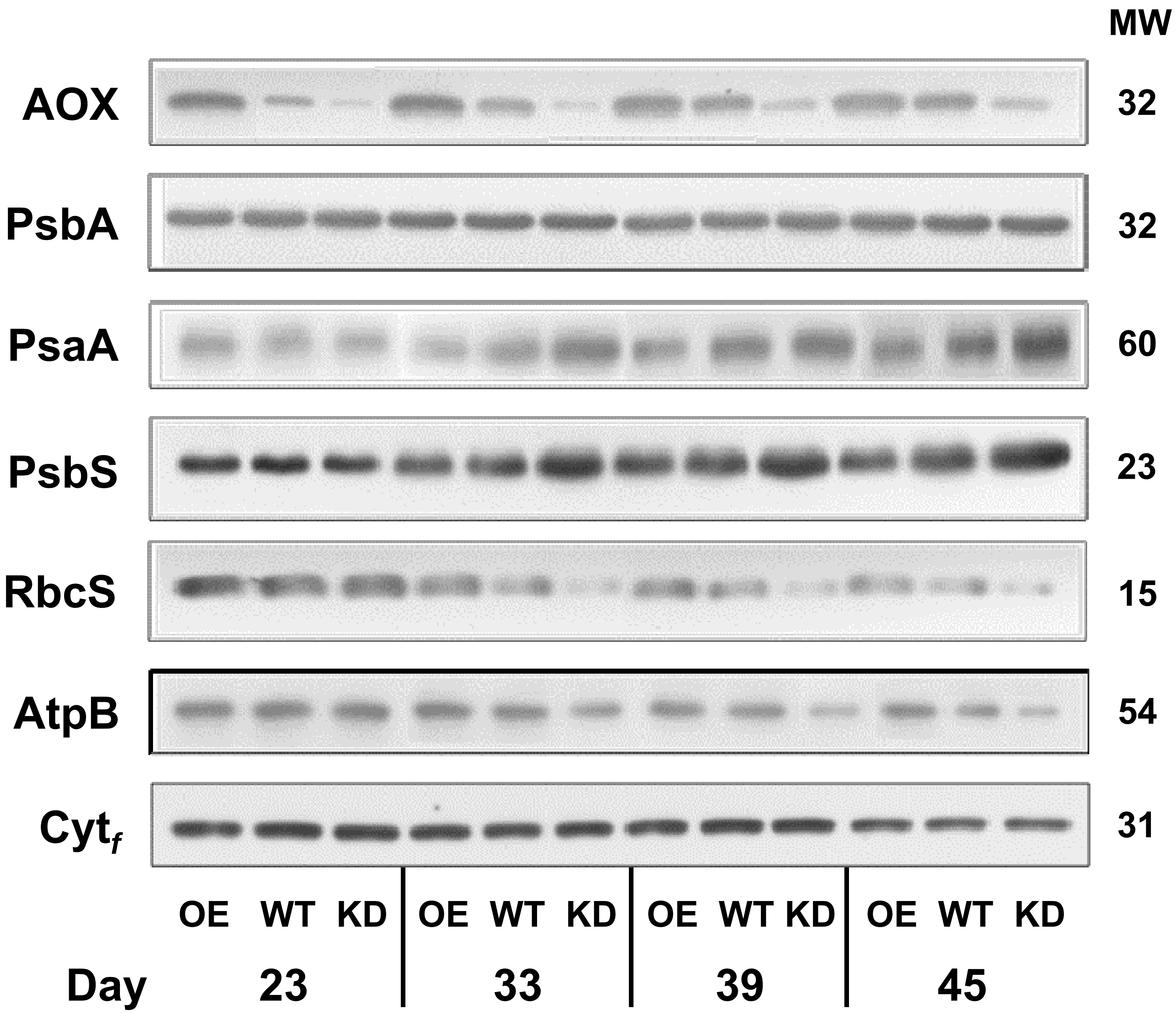

Supplementary Figure S13

Supplementary Figure S13 │ Representative immunoblots for AOX and several photosynthesis-related proteins at different times during a prolonged water deficit. WT, wild-type. KD, AOX knockdowns (combined RI9 and RI29). OE, AOX overexpressors (combined B8 and B7). See text for further details. MW, molecular weight.

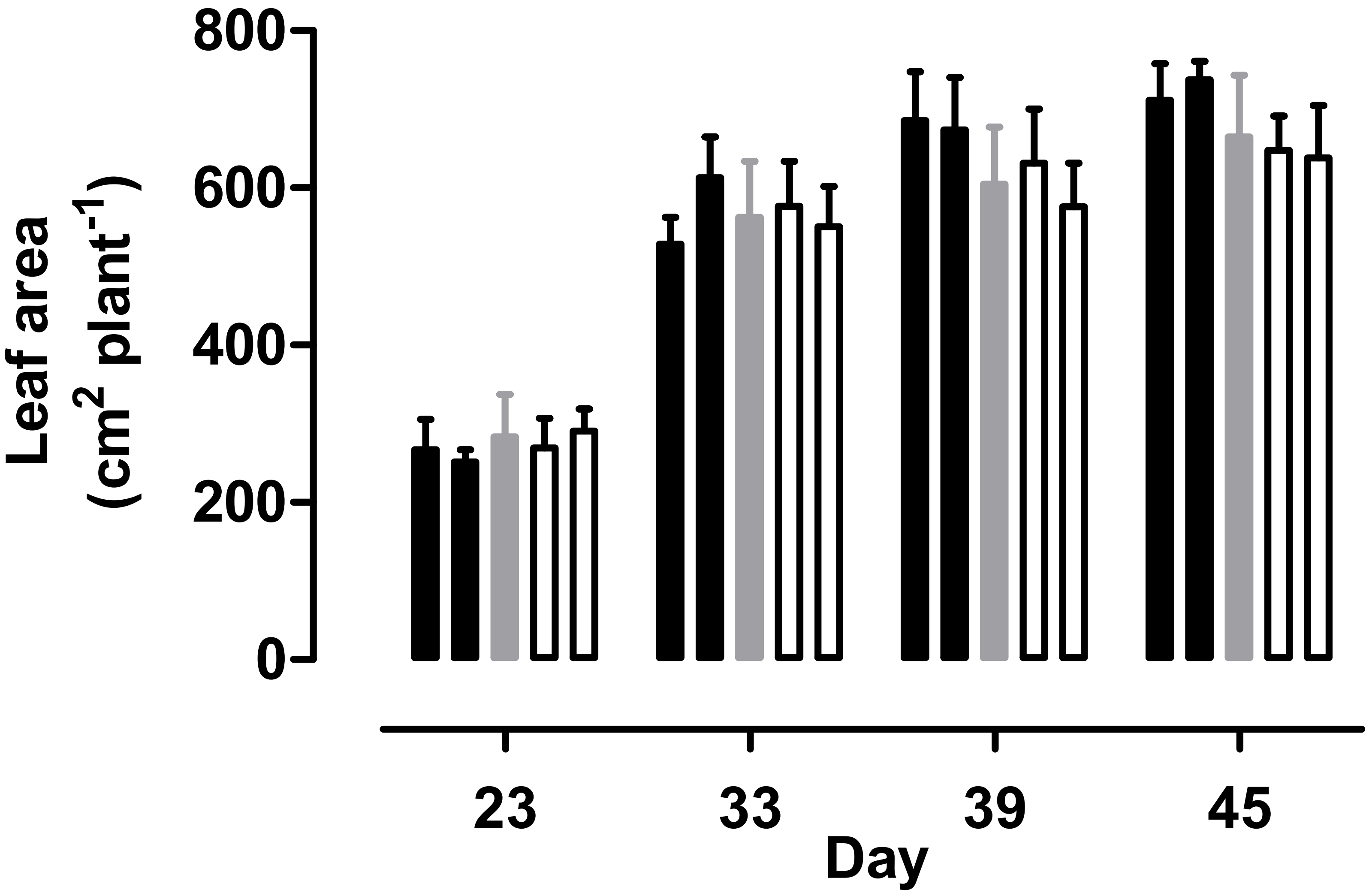

Supplementary Figure S14

Supplementary Figure S14 │ Total tobacco plant leaf area at different times during a prolonged water deficit. Data are presented for WT (grey bar), AOX overexpressors (B8, left closed bar; B7, right closed bar) and AOX knockdowns (RI9, left open bar; RI29, right open bar) and represent the mean $\pm$ SE of three independent experiments (n=3). Within each data set, there are no significant differences across plant lines.

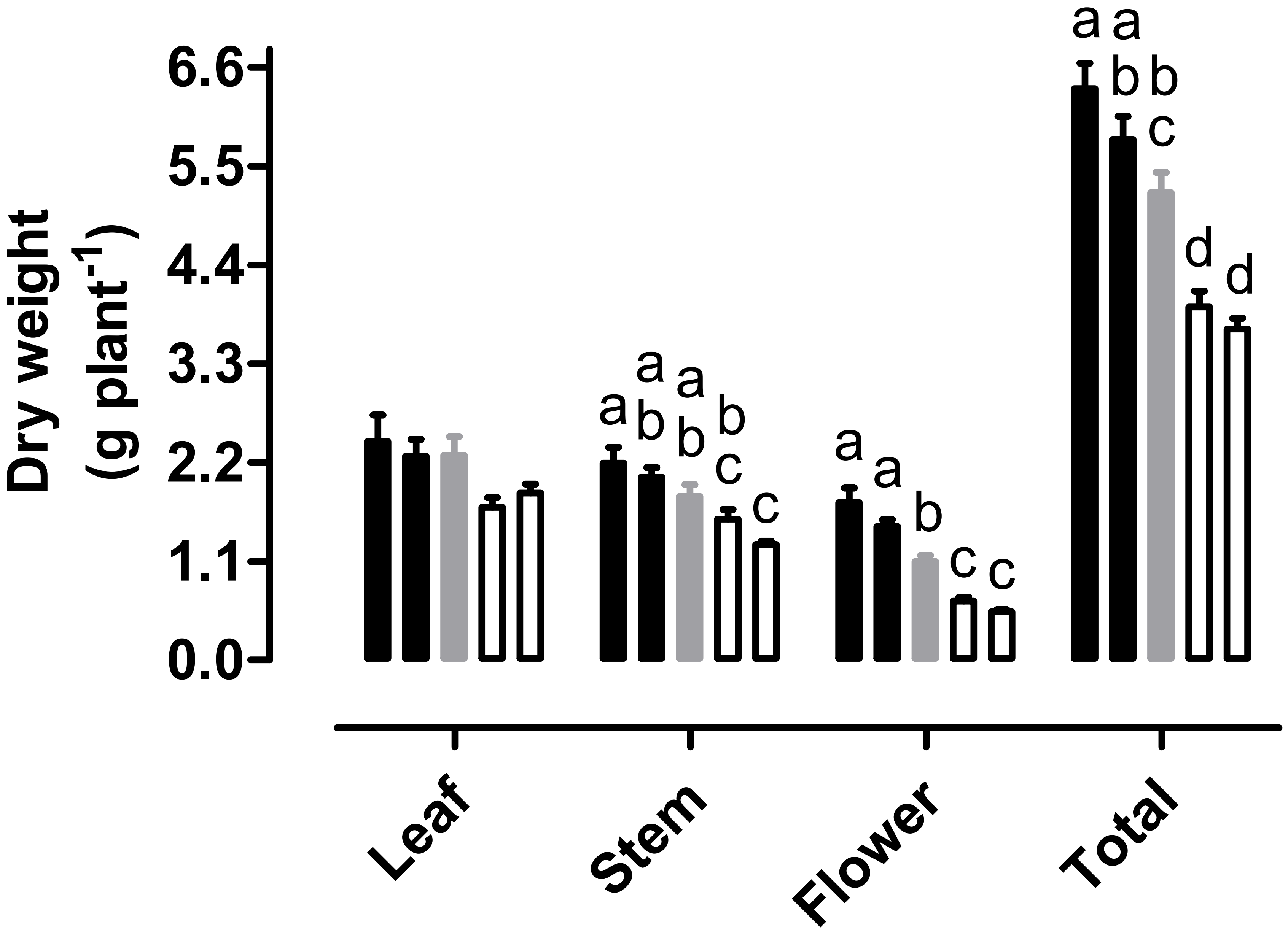

Supplementary Figure S15

Supplementary Figure S15 │ Tobacco plant DW after a prolonged water deficit. Data are shown for leaves, stems, reproductive tissue (flowers and seed pods) and total shoot, harvested at day 45. Data are presented for WT (grey bar), AOX overexpressors (B8, left closed bar; B7, right closed bar) and AOX knockdowns (RI9, left open bar; RI29, right open bar) and represent the mean $\pm$ SE of six independent experiments (n=6). Within each data set, plant lines not sharing a common letter are significantly different from one another ($P<0.05$). In data sets without letters, there are no significant differences across plant lines.
